# Strategies of Designing High-Efficiency Electrolyte Additives for Aqueous Magnesium Batteries: A Review

**DOI:** 10.1007/s40820-026-02299-1

**Published:** 2026-07-27

**Authors:** Yulong Wu, Darya Snihirova, Yibing Zhang, Xiaohui Zeng, Wen Xu, Linqian Wang, Daniel Höche, Sviatlana V. Lamaka, Mikhail L. Zheludkevich

**Affiliations:** 1 Institute of Surface Science, Helmholtz-Zentrum Hereon, Max-Planck Str. 1, 21502 Geesthacht, Germany; 2 School of Materials Science and Engineering, Hebei University of Technology, Tianjin, 300000 People’s Republic of China; 3 Institute of Materials Science, Kiel University, 24143 Kiel, Germany

**Keywords:** Aqueous Mg-air battery, Electrolyte additive, Self-discharge, Machine learning

## Abstract

With the development of next-generation energy storage systems focusing on green, safe, and sustainable devices, aqueous magnesium (Mg)–air batteries have emerged as promising candidates owing to the intrinsic merits of the Mg metal anode, including low cost, large volumetric capacity, a highly negative electrode potential, and excellent safety. However, the high self-discharge rate and formed discharge products substantially impair the practical discharge performance of Mg anodes with poor anode utilization efficiency and low cell voltage. While anode alloying and electrolyte additive strategies can help mitigate this issue, designing highly efficient electrolyte additives that are compatible with the established anode remains a challenge. This review summarizes recent progress toward understanding the role of electrolyte additives for aqueous Mg batteries, provides insights into the discharge mechanism of Mg-based anode materials in different types of additive-containing electrolytes, and offers strategies for designing high-efficiency mixtures of electrolyte additives. Moreover, a highly promising direction of AI-supported and robotic workflows for the future fast design of advanced electrolyte additives for aqueous Mg batteries is discussed.

## Highlights

- This review provides a systematic summary of aqueous Mg–air electrolyte additives, offering structured data to support future machine learning screening, especially for binary additive systems.
- This work emphasizes the significant influence of oxygen reduction reaction-induced oxygen corrosion on Mg anode utilization, particularly for clean Mg anodes in additive-containing electrolytes.
- This article proposes a clear design concept for binary additives, enabling rational rather than empirical combinations and guiding the development of high-performance hybrid additives for aqueous Mg–air batteries.

## Graphical abstract

**Figure:**
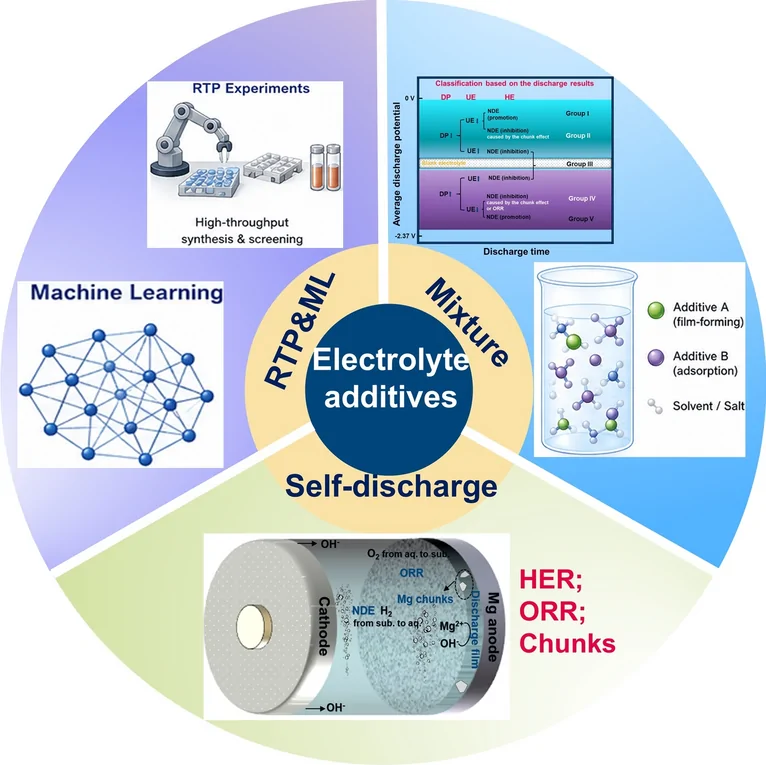

## Introduction

### Aqueous Mg–Air Batteries

**Fig. 1:**
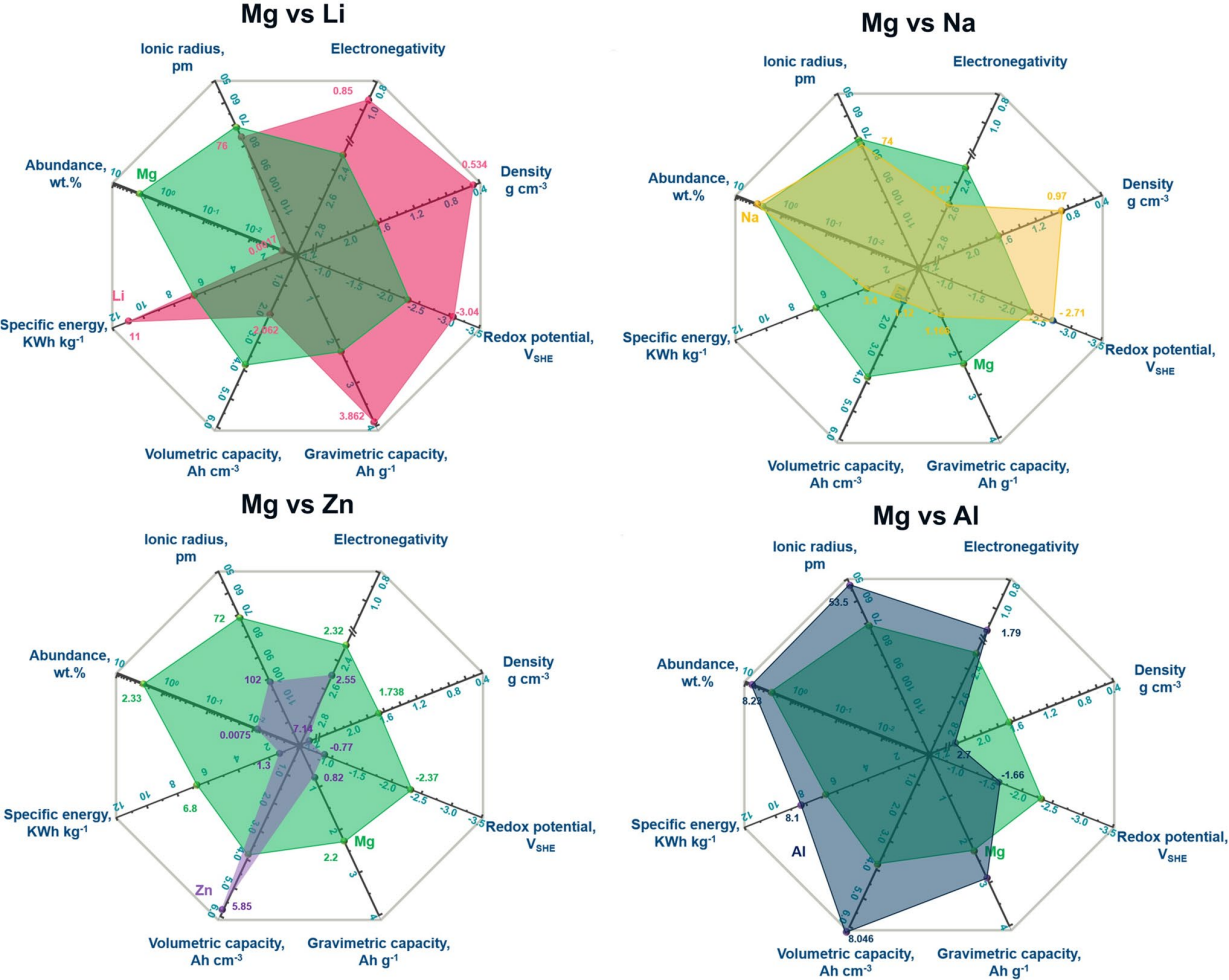
Comparison of traditional and emerging metallic anodes (Li, Zn, Na, Al, and Mg) for energy storage applications. Elemental abundance is expressed as weight fraction in Earth’s crust

**Fig. 2:**
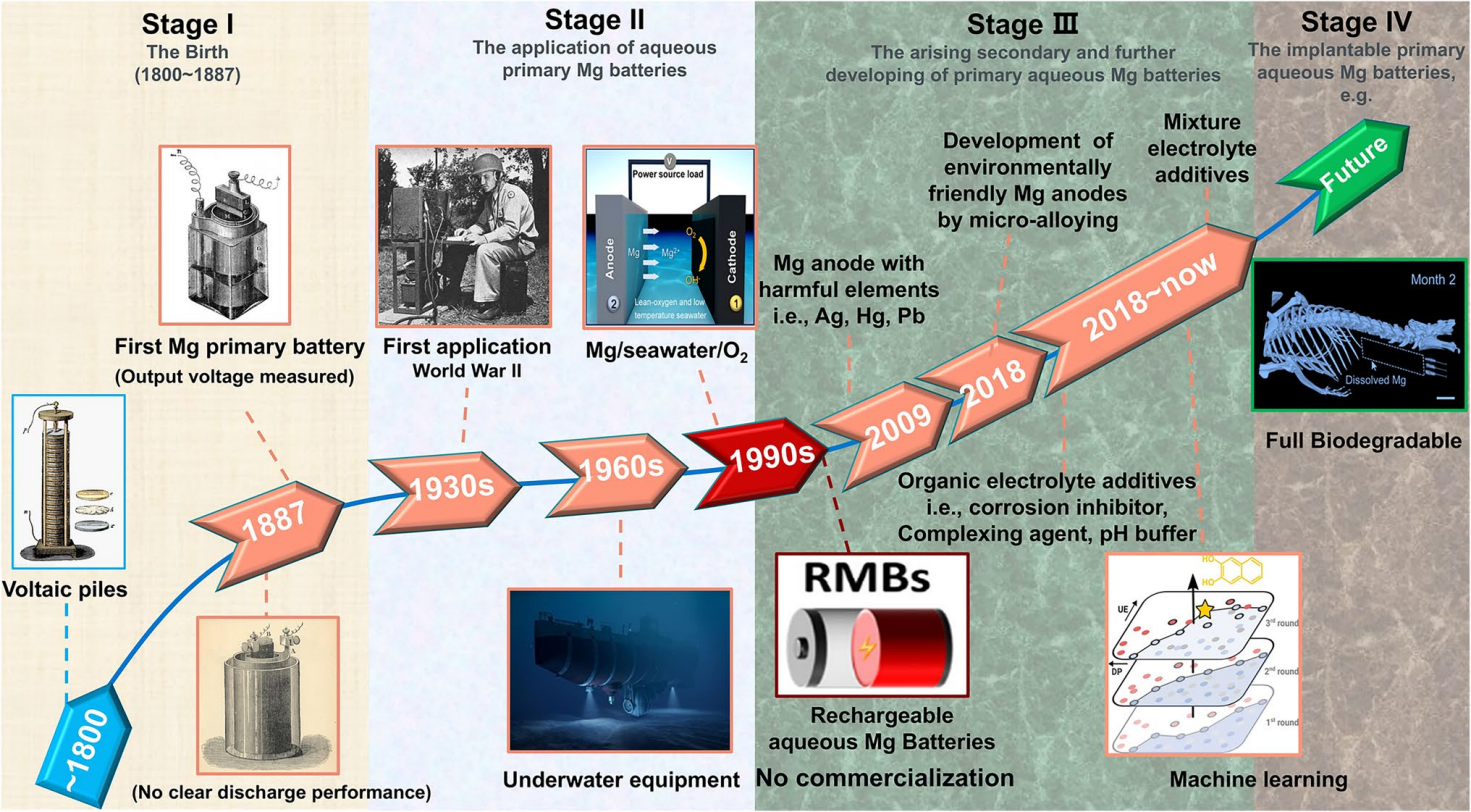
Milestones in the development of aqueous Mg batteries

Metal–air batteries have attracted extensive attention as promising candidates for next-generation energy storage systems due to their intrinsic safety, low cost, environmental friendliness, and high ionic conductivity of aqueous electrolytes. These advantages have inspired increasing interest in exploring various metal anodes for battery [1–5]. Figure 1 provides a summary of key parameters for various candidate anode metals in energy storage applications, highlighting system with metallic magnesium (Mg) anodes for its well-rounded performance [6–9]. Among various Mg battery systems, aqueous Mg–air batteries are the most widely studied. This is attributed to their abundant raw materials, low cost, highly negative redox potential (−2.37 V vs. SHE), and large theoretical specific energy density (6.8 kWh kg^−1^) [10]. The development history of aqueous batteries with Mg alloys as anodes is shown in Fig. 2. The theoretical advantages of Mg as an anode for primary batteries were undoubtedly recognized even in the days of Volta. As early as 1887, the metallic Mg as a primary battery anode was proposed by G. Hein [11]. Then, he modified the Leclanché cell consisting of an Mg anode, a MnO_2_ cathode, and an aqueous MgCl_2_ or MgSO_4_ electrolyte, which marked the first well-documented aqueous primary Mg battery [12, 13]. The first application of aqueous primary Mg batteries was in the early 1930s as communication equipment and beacons for World War II [14]. In the 1960s, the concept of aqueous primary Mg–air batteries gained attraction as research teams at the U.S. Naval Research Laboratory (NRL) began researching their use in submarines and other underwater equipment [15]. In 1993, the Norwegian Defence Research Establishment (FFI, Norway) developed the Mg/seawater/O_2_ cell specifically for autonomous underwater vehicles (AUVs) [16]. Shortly thereafter, a collaborative initiative involving FFI, the Direction Generale Armament (DGA, France), and the Bassin d’Essais des Carenés (BEC, France) resulted in the design of the CLIPPER battery system specifically for AUV applications [17]. This innovative system enabled the underwater vehicle to travel up to 1600 nautical miles at a speed of approximately 2 m s^−1^. Prior to 2018, progress in aqueous Mg batteries predominantly focused on adding inorganic compounds as electrolyte additives in water, including nitric acid, nitrites, sulfates, and sodium chloride [18–21]. In addition, most of the Mg anodes in service for practical applications contain environmentally hazardous alloying elements, i.e., Hg, Pb [22–25]. This is because these elements can assist Mg anodes in delivering high cell voltage during short-term discharge [26] by activating the anode surface. Since 2018, research on aqueous Mg batteries has advanced rapidly, with a strong focus on developing innovative environmentally friendly Mg anodes and optimizing electrolytes by adding organic electrolyte additives [27, 28]. For instance, Deng et al. [29–31] proposed a microalloying strategy for Mg anode design, with the lean Mg-0.15 Ca alloy emerging as a notable achievement. This alloy has been lauded as “stainless magnesium” under open-circuit potential (OCP) conditions and exhibits both high cell voltage and promising anode utilization efficiency under polarization conditions [31]. In addition to designing next-generation environmentally friendly Mg anode by microalloying, multi-objective machine learning strategies have been employed for the active design and efficient screening of Mg anodes [32, 33]. With the rapid advancement of Mg anode materials in recent years, the development of complementary electrolyte additives has increasingly focused on organic compounds, including corrosion inhibitors [34, 35], Fe^2+/3+^ and Mg^2+^ complexing agents [27, 36, 37], pH buffers [38], and mixed additives [39, 40]. To enable efficient large-scale screening of aqueous Mg battery additives, Würger et al. [41] adopted active learning adaptive experimental design workflow based on a quantitative structure–property relationship (QSPR) model. This approach facilitated multi-objective optimization in an AI-assisted search for electrolyte additive molecular descriptors and respective chemical formulations that simultaneously enhance discharge potential (DP) and utilization efficiency (UE). Based on this, Wu et al. [42] discovered 2,3-dihydroxynaphthalene as a powerful additive for aqueous Mg–air battery. When introduced into a 3.5 wt% NaCl solution, the electrolyte achieved in a laboratory-made (Mg-0.2Ca)–air battery the highest specific energy reported in tests to date among Mg–air batteries. In the past five years, fully biodegradable implant batteries have become a research hotspot as well. Preliminary research results have been achieved on primary batteries aqueous designed with Mg anodes [43–48]. Leveraging the compatibility advantages of Mg anodes, fully biodegradable aqueous Mg batteries are emerging as one of the promising future directions, particularly for use as implantable primary devices within the body. In addition, Mg–air batteries show great potential as power sources for remote sensing, marine transportation, pipeline and tank monitoring systems, hazardous environments, space backup power systems, and other specialized applications. The combination of lightweight design properties, ease of use, material abundance, and non-toxicity makes Mg-based batteries especially attractive for these niche applications.

### Challenges of Aqueous Mg Batteries

#### Conventional Challenges of Aqueous Mg Batteries

The electrochemical reactions in an electrochemical cell with Mg anode in the 3.5 wt% NaCl solution (neutral alkaline environments) during the discharging process are listed in the following equations [49, 50]:

1 $${\mathrm{Hydrogen}}\;{\mathrm{evolution}}\;{\text{reaction }}\left( {{\mathrm{HER}}} \right)\!:\,{\text{Mg + 2H}}_{{\mathrm{2}}} {\mathrm{O}} \to {\mathrm{Mg}}\left( {{\mathrm{OH}}} \right)_{{\mathrm{2}}} {\text{ + 2H}}_{{\mathrm{2}}}$$

2 $${\mathrm{Oxygen}}\;{\mathrm{reduction}}\;{\text{reaction }}\left( {{\mathrm{ORR}}} \right){\text{: 2Mg + O}}_{{\mathrm{2}}} {\text{ + 2H}}_{{\mathrm{2}}} \to {\mathrm{O}}_{{\mathrm{2}}} {\mathrm{Mg}}\left( {{\mathrm{OH}}} \right)_{{\mathrm{2}}}$$

**Fig. 3:**
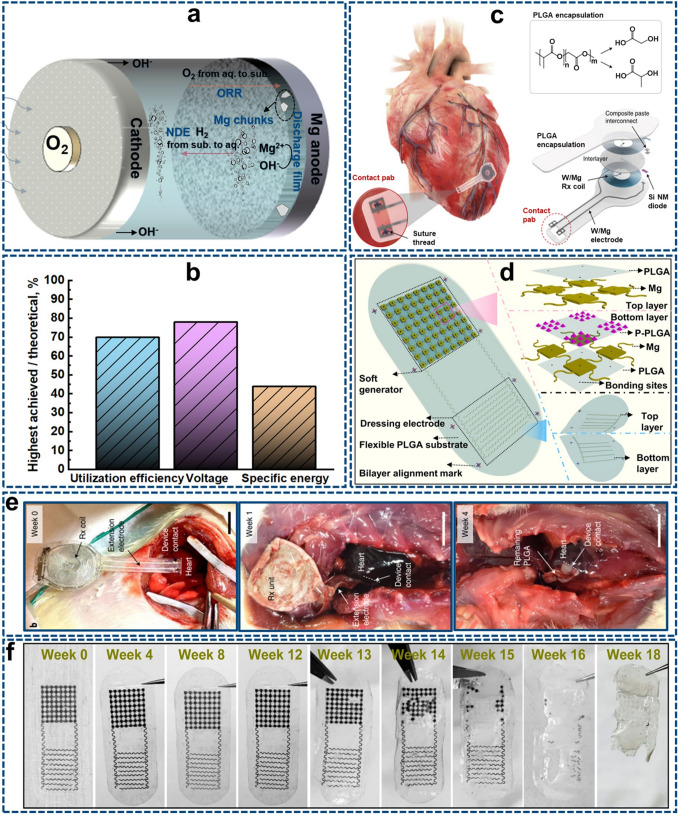
**a** Working mechanisms schematic of self-discharge of Mg anode in the aqueous electrolyte **b** percentage of the highest achieved values of utilization efficiency, cell voltage, and specific energy to their theoretical value. **c, d** Schematic diagram of components for two types of primary Mg implant batteries. **e, f** Biodegradation challenge of the above-motioned two primary Mg implant batteries. **c, e** Reproduced with permission from Ref. [45] Copyright 2021, Springer Nature. **d, f** Reproduced with permission from Ref. [55] Copyright 2021, National Academy of Sciences.

Figure 3a presents a schematic illustration of the working mechanisms, highlighting the self-discharge processes occurring on the Mg anode in aqueous electrolytes. During discharge, the parasitic anodic HER, resulting from the negative difference effect (NDE), occurs on Mg anode, markedly decreasing the anode UE. In addition to HER, the parasitic ORR on Mg anode has increasingly been recognized for its contribution to anode UE loss in the discharge behavior of aqueous primary Mg batteries [38]. The chunk effect, referring to the detachment of small non-oxidized Mg anode metallic fragments that cannot discharge effectively due to loss of electrical contact with the anode, is another critical challenge that significantly reduces anodic UE. Besides the negative effect on anodic UE by NDE, ORR, and chunks, the precipitated insoluble discharge product film increases internal battery resistance, thereby reducing the DP. As a result, the anode UE rarely surpasses 60%, the cell voltage typically falls below 1.6 V, which is approximately 70% of its theoretical value (Fig. 3b). The specific energy, even when calculated under relatively favorable discharge conditions (e.g., DP ≈ 1.6 V and UE ≈ 60%), remains below 30% of its theoretical maximum [49, 51–54].

#### Emerging Challenges in Bio-Related Application

Beyond the challenges outlined above, the application of aqueous Mg batteries in specialized scenarios faces additional obstacles. Bio-related applications are discussed separately due to their growing importance as a promising future research direction. Nowadays, the implantable aqueous primary Mg batteries are emerging as a pivotal research area, playing a crucial role in the development of cutting-edge medical devices [43]. These technologies enable seamless interaction with biological systems, advancing the diagnosis, monitoring, and treatment of diverse health conditions. Aqueous primary Mg batteries as implantable devices can be designed with the function of being fully biodegradable which offers a potential solution to avoid the additional costs and wounds associated with a second surgery. Figure 3c, d illustrates the schematic assembly of a miniature primary cell with Mg as an anode electrode, specifically designed for implantable devices. These batteries, classified as aqueous Mg batteries, are activated by body fluids as electrolyte. Figure 3e presents images of the cell, assembled in Fig. 3c, implanted in a rat model, highlighting various stages of bioresorption over 4 weeks [45]. Figure 3f presents optical images of the fracture electrostimulation device shown in Fig. 3d, immersed in phosphate-buffered saline solution at 37 °C, demonstrating its natural degradation process over an 18-week period [55]. The operational lifespan of the battery might be prematurely curtailed due to varying degrees of degradation observed in both the Mg anode and the organic shell of the implanted battery device. Until now, no studies have specifically addressed the premature termination of operation in such implanted devices caused by the degradation of non-critical components during employment. Based on the effective service time (${T}_{s}$) and the full degradation time (${T}_{d}$) of the Mg batteries, a new concept should be proposed and emphasized which is the real implantable battery utilization efficiency (*BUE*). The BUE was determined using the following expression:

3 $$BUE = \frac{{T_{s} }}{{T_{d} }} \times 100\%$$

Herein, the higher value of *BUE* signifies a shorter degradation duration of residual component retention in the body after the battery ceases to function. This can reduce the delayed organ recovery associated with the prolonged retention of ineffective battery components. Beyond the low *BUE* challenge, the excessive hydrogen evolution can lead to gas pocket formation, while local pH elevation may cause severe cytotoxicity, undermining both safety and biocompatibility.

## Approaches to Improving the Discharge Behavior of Mg Anodes

**Fig. 4:**
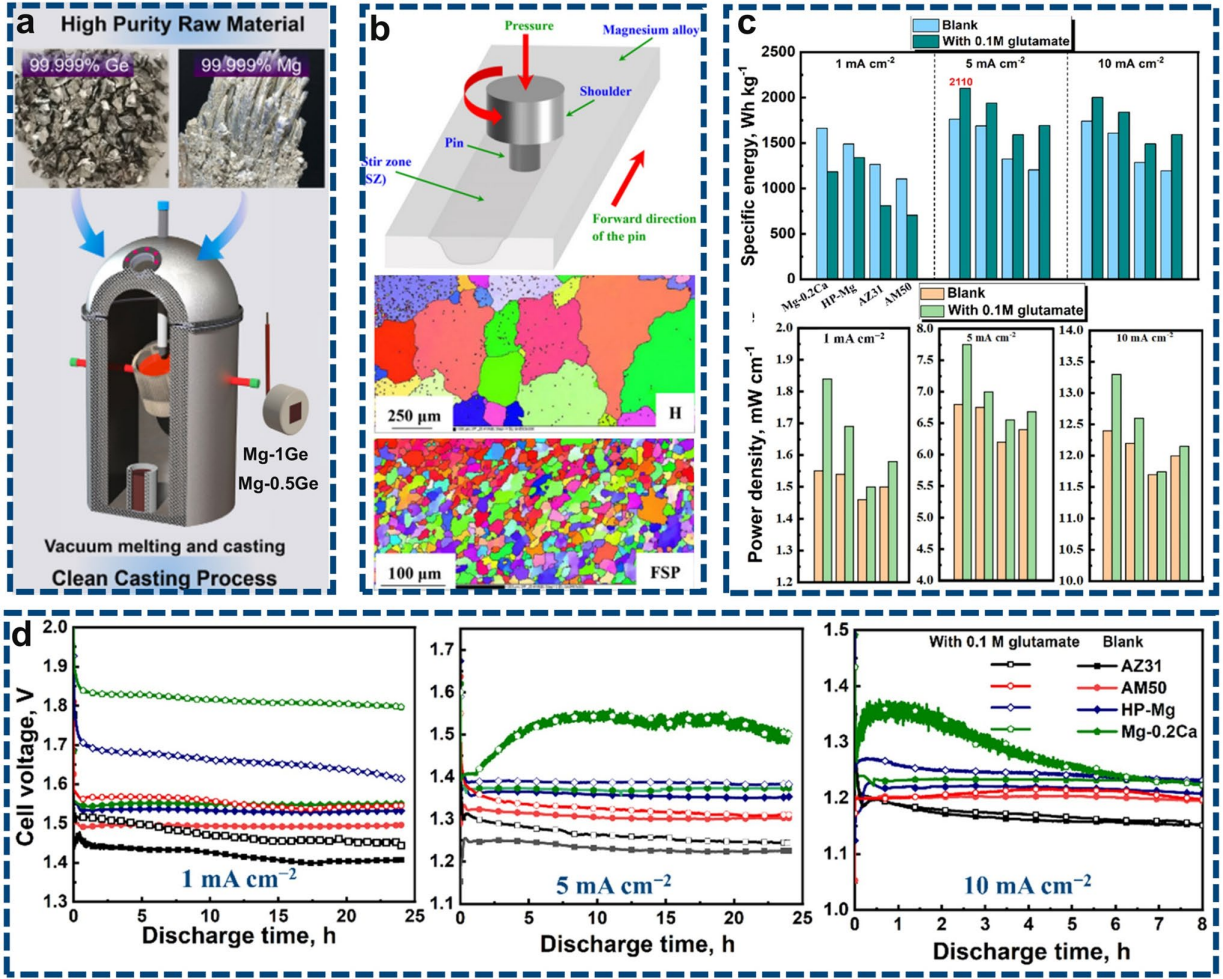
**a** Schematic diagram of the fabrication procedure for the UHP Mg-Ge anodes. Reproduced with permission from Ref. [59] Copyright 2024, Elsevier. **b** Trajectory of the tool during friction stir processing (FSP), and SEM images of as-cast Mg–Al-Sn-RE homogenized alloy (H) and the alloy after FSP. Reproduced with permission from Ref. [62] Copyright 2024, Elsevier. **c, d** Specific energy, power density, and cell voltage of Mg anodes in glutamate-containing electrolytes, the applied current is 5 mA cm^−2^. Reproduced with permission from Ref. [38] Copyright 2025, Elsevier.

The discharge performance of primary aqueous Mg batteries has been gradually improved through (i) designing advanced Mg anodes, and (ii) regulating the Mg–electrolyte interface using electrolyte additives [56–58]. From the perspective of anode development, microalloying has proven to be an effective strategy, as shown in Fig. 4a, Chen et al. [59] produced a class of lean ultra-high-purity (UHP) Mg-Ge anodes for Mg–air batteries through microalloying techniques, achieving a high discharge voltage of 1.69 V (0.5 mA cm^−2^) and a high energy density of 2.27 kWh kg^−1^ (20 mA cm^−2^) in full-cell battery using MnO_2_/C as cathode (O_2_) catalyst. The superior performance of the lean Mg–Ge alloy system arises from the combined effects of ultra-low impurity content and the multifunctional role of Ge, which increases the alloy’s open-circuit potential through Mg_2_Ge particle formation and forms a protective Ge-enriched surface layer that mitigates self-discharge. In addition to alloy design, severe plastic deformation and advanced thermo-mechanical processing techniques have also been employed to refine microstructure and boost anode activity. However, these benefits are not universal. For example, in Mg–Ca and related micro-alloyed systems, the influence of extrusion and other hot deformation processes on corrosion behavior can be complex. Although such processing typically leads to grain refinement and increased dislocation density, it also alters secondary phases, intermetallic distributions and galvanic coupling behavior, which usually brings an adverse effect to anode discharge performance [60, 61]. As shown in Fig. 4b, Liu et al. [62] demonstrated that friction stir processing (FSP) treated Mg alloys exhibit significantly enhanced discharge performance owing to improved grain refinement and reduced microgalvanic heterogeneity. From the perspective of electrolyte additives development, several individual electrolyte additives—such as glutamate [38]—have been reported to simultaneously enhance the specific energy, power density, and cell voltage of lean Mg-0.2Ca anode, as shown in Fig. 4c, d. However, prior studies also reveal that the compatibility between different classes of electrolyte additives and various Mg-based anodes remains insufficiently understood. This knowledge gap underscores the necessity of elucidating the interfacial reaction pathways that govern discharge behavior. A deeper mechanistic understanding across different anode-additive combinations are essential for establishing universal design principles and developing next-generation electrolyte additive systems capable of delivering both high DP and UE.

**Fig. 5:**
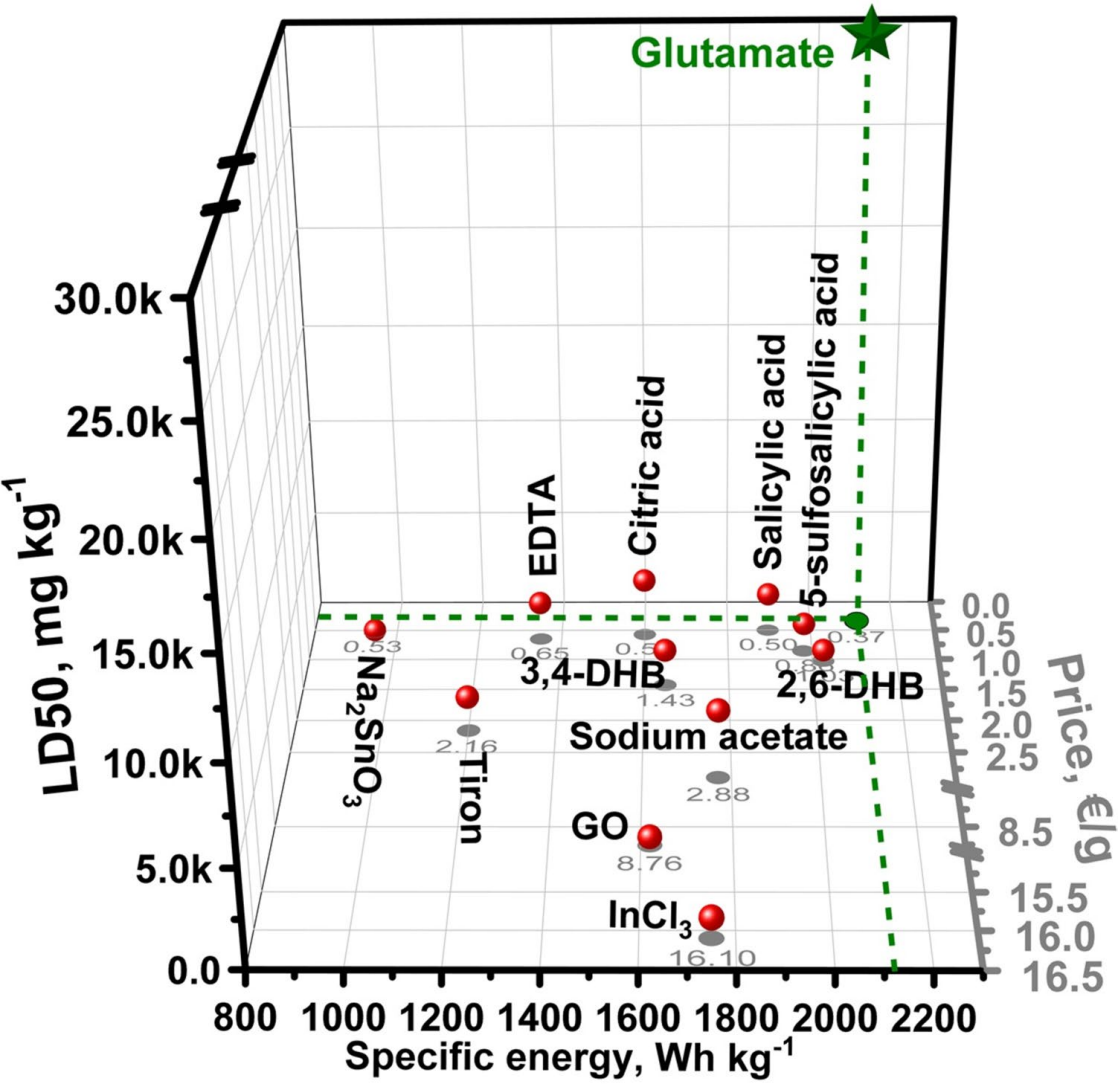
Comparative summary of market price, toxicity (LD50), and specific energy (applied current density is 5 mA cm^−2^) of electrolyte additives reported for aqueous Mg battery systems. Reproduced with permission from Ref. [38] Copyright 2025, Elsevier

When selecting electrolyte additives, considerations related to economic feasibility, environmental compatibility, and operational safety are essential for advancing aqueous Mg battery technologies toward practical implementation. Early studies predominantly explored simple inorganic salts (e.g., NaNO_3_, Na₂SO_4_, NaVO_3_, and Na_3_PO_4_), which are inexpensive, readily accessible, and generally benign, but their ability to enhance electrochemical performance is limited. As research evolved, efforts shifted toward more sophisticated organic additives and engineered nanomaterials (i.e., GO), which can deliver improved performance but often come with higher material costs and more complex synthesis routes. Figure 5 provides a comparative assessment of several representative high-performing additives reported in the literature, including their market price, toxicity (LD50), and specific energy at an applied current density of 5 mA cm^−2^. Considering material affordability, environmental and toxicological profiles, and discharge performance in a comprehensive manner, glutamate emerges as one of the most promising electrolyte additives identified to date.

## Electrolyte Systems and Their Impact on Mg Anode

Previous studies have systematically evaluated the discharge performance of Mg anodes in various chloride-free electrolytes, including nitrate/nitrite, phosphate, and sulfate systems [49, 63]. In conclusion, these electrolytes suppress the self-discharge of Mg anodes and therefore enhance their UE by avoiding the aggressive chloride-induced pitting mechanism [64–67]. Mechanistically, the absence of Cl^−^ minimizes localized attack, leading to more uniform anode dissolution. However, these advantages are accompanied by significant drawbacks: chloride-free systems often promote the formation of dense, passivating discharge product layers (e.g., Mg(OH)_2_, Mg_3_(PO_4_)_2_, MgSO_4_·xH_2_O), which hinder ion transport, increase interfacial resistance, and consequently lower the operating cell voltage especially at higher current densities [68, 69]. For Mg-based primary batteries, the most traditional and widely used electrolytes are chloride-containing systems, including 3.5 wt% NaCl solutions and seawater, because they reliably activate the Mg anode and provide high discharge voltages under practical conditions. These chloride-based media have long served as the baseline for evaluating new electrolyte formulations. In contrast, chloride-containing electrolytes—particularly NaCl—induce a more porous and easily detachable Mg(OH)_2_/MgO layer, resulting in higher discharge voltages and more stable anode activation. Because NaCl also offers advantages such as low cost, environmental benignity, high solubility, and well-understood chloride chemistry, it has become the most widely adopted electrolyte for practical Mg-based primary systems. Therefore, the present review focuses specifically on NaCl-based electrolytes and the mechanistic factors governing their performance. Therefore, in this review, we focus on the discussion of electrolytes in the NaCl system.

Before discussing the effects of aqueous electrolytes containing various additives on Mg anode self-corrosion and the potential roles of different additives in regulating these processes, the following aspects related to NDE are summarized. The NDE in Mg anodes originates from the nonlinear coupling between anodic Mg dissolution and the hydrogen evolution reaction (HER), where the overall anodic current decreases with increasing overpotential. However, this behavior is strongly dependent on interfacial kinetics, which can be significantly modified by electrolyte additives. From a kinetic perspective, the influence of additives on NDE can be categorized into three main pathways. First, additives such as complexing agents can modify Mg^2^⁺ solvation and interfacial charge transfer, thereby altering HER kinetics and shifting the balance between cathodic hydrogen evolution and anodic dissolution. Second, pH buffers regulate the local alkalization process and influence the stability of Mg(OH)_2_ surface films, which directly affects the dynamic passivation–depassivation cycle. Third, corrosion inhibitors tend to adsorb on active dissolution sites, leading to heterogeneous current distribution and suppression of localized anodic hotspots. These distinct kinetic effects determine whether the NDE is weakened or enhanced, indicating that NDE is not solely an intrinsic property of Mg but a strongly electrolyte-dependent interfacial phenomenon.

### Sodium Chloride Electrolyte System

In NaCl aqueous electrolytes, the efficiency loss of Mg anode involves two processes: Self-corrosion and self-discharge. The difference between self-corrosion and self-discharge lies in their operating conditions. Self-corrosion occurs under open-circuit conditions (OCP) during the off-discharge periods of aqueous Mg batteries, whereas self-discharge takes place under polarization during discharge [70–73]. Both processes typically involve the HER and ORR. In contrast to self-corrosion at OCP, self-discharge involves highly nonuniform anodic dissolution, which provides more opportunities for the formation of metallic Mg chunks [54]. Those Mg detached Mg chunks cannot participate in discharge, resulting in a reduce in anode UE. This phenomenon is known as the chunk effect.

**Fig. 6:**
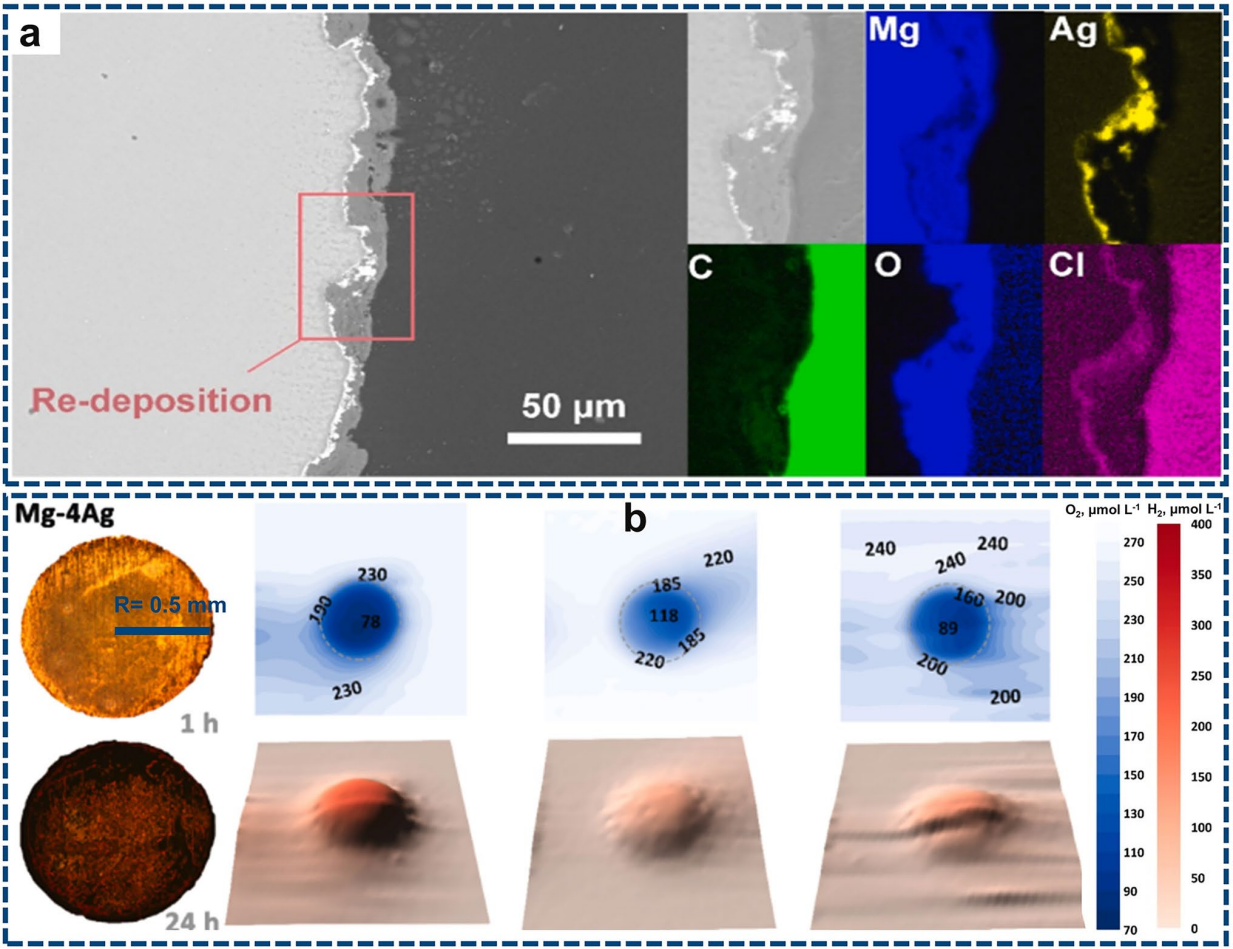
**a** Cross-section images and EDS analysis of Mg-4Ag in 0.85 wt% NaCl electrolyte. **b** Visual appearances, and the distribution of local concentration of O_2_ measured 50 µm above the surface of Mg-4Ag with the immersion in 0.85 wt% NaCl electrolyte under hydrodynamic condition. Reproduced with permission from Ref. [74] Copyright 2024, Elsevier

The ORR-driven self-discharge has often been overlooked in NaCl electrolytes, primarily because the relatively thick Mg(OH)_2_ discharge product layer is assumed to hinder the diffusion of dissolved oxygen to the anode surface. This assumption indeed applies to most conventional Mg anodes. However, it does not necessarily hold for certain Mg alloys, where the presence of specific alloying elements is involved. For example, as shown in Fig. 6a, Wang et al. [74] discovered that ORR can also be very fast in the case of a thick corrosion product layer for Mg-Ag alloy. This is attributed to the Ag-redeposits spreading through the interfacial Mg(OH)_2_ layer, which promoted ORR (Fig. 6b). As a result, the self-discharge mechanisms of Mg alloys during discharge in NaCl electrolyte differ according to the alloying elements incorporated during alloying, primarily because such elements modify the kinetics and pathways of both anodic dissolution and cathodic reactions. Therefore, the reactions of specialized Mg anodes, i.e., Mg–Ag-based anodes, should be thoroughly considered to gain a deeper understanding of the discharge mechanisms, with particular attention to the influence of redeposition of alloying elements on discharge properties.

### Sodium Chloride Electrolyte System with Additives

Electrolyte additives for aqueous Mg batteries refer to a class of compounds that, when present in a cell environment at an appropriate concentration and in the right form, can inhibit self-discharge of anodes or/and boost the cell voltage of batteries. Therefore, the employment of electrolyte additives in the 3.5 wt% NaCl solution is also one of the effective strategies to improve the discharge performance of aqueous Mg batteries [67, 75]. In the literature, electrolyte additives can be further classified based on the discharge mechanism: complexing agents [27, 36, 76], pH buffers [38], corrosion inhibitors [35], and surfactants [34, 77].

**Fig. 7:**
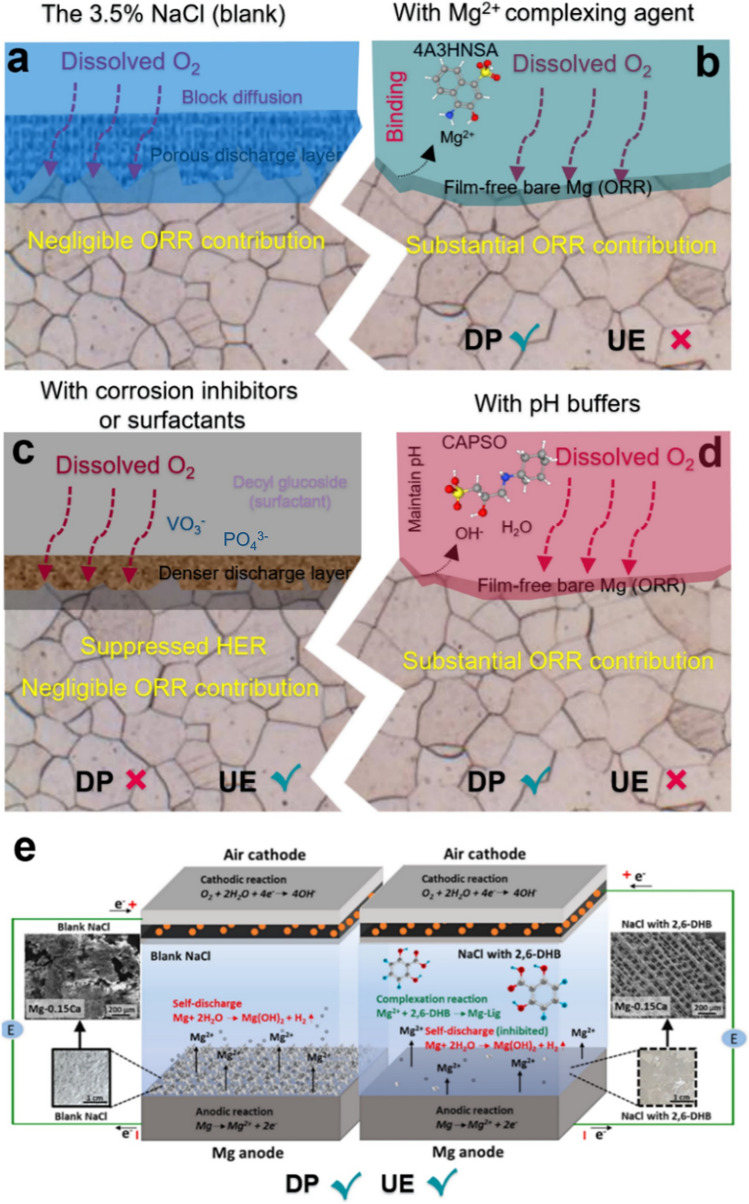
Schematic illustration of discharge mechanism of Mg anode in various additive-containing electrolytes. **a** In the blank electrolyte, **b** In the blank electrolyte with Mg^2+^ complexing agents, **c** In the blank electrolyte with pH corrosion inhibitors and surfactants, **d** In the blank electrolyte with pH buffer agents, **e** In the blank electrolyte with 2,6-DHB. Reproduced with permission from Ref. [78] Copyright 2022, Elsevier

As illustrated in Fig. 6a, in the blank electrolyte, the Mg anode undergoes rapid anodic dissolution accompanied by extensive hydrogen evolution and discharge products (MgO & Mg(OH)_2_). The discharge products effectively prevent the diffusion of dissolved oxygen in the electrolyte, inhibit the ORR, and simultaneously have a negative effect on the DP. In Fig. 7b, d, the electrolyte additives that complexing agents and pH buffers can regulate the interfacial concentrations of Mg^2^⁺ and OH⁻, respectively, thereby suppressing or delaying the formation of Mg(OH)_2_-based discharge product films. Consequently, they typically lead to higher DP due to facilitated ionic transport and surface renewal, but at the expense of lower UE. This is because their strong interfacial activity often accelerates HER and the relatively clean anode surface cannot effectively prevent the diffusion of dissolved oxygen in the electrolyte, thereby accelerating the ORR as well. In contrast, as shown in Fig. 7c, the electrolyte additives that corrosion inhibitors and surfactants act mainly as film-forming promoters that mitigate the self-discharge of Mg anodes through mechanisms such as passivation, precipitation, or interfacial adsorption. As a result, these additives generally provide improved UE, but their tendency to stabilize or thicken the surface film often leads to reduced DP.

It is important to note that although the formation of a discharge product film consistently exerts a detrimental effect on the discharge voltage, its influence on anode UE is more complex. In general, only when the film becomes sufficiently compact to serve as an effective physical barrier under anodic polarization can it suppress the HER and consequently enhance the UE. To simultaneously enhance both DP and UE, electrolyte additives should be capable of suppressing self-discharge while preserving a relatively clean Mg surface. As illustrated in Fig. 7e, Wang et al. [78] demonstrated that 2,6-dihydroxytbenzoate (2,6-DHB) effectively inhibits parasitic HER while preventing excessive accumulation of discharge products, thereby achieving concurrent improvements in DP and UE. Although a few of such examples exist, electrolyte individual additives capable of simultaneously delivering both high DP (maintaining a clean anode surface) and high UE remain exceedingly rare. Therefore, it is necessary to gain deeper insight into the underlying working mechanisms, particularly the influences of HER, ORR, and chunk effect—across different categories of electrolyte additives.

#### Role of Additives on HER

**Fig. 8:**
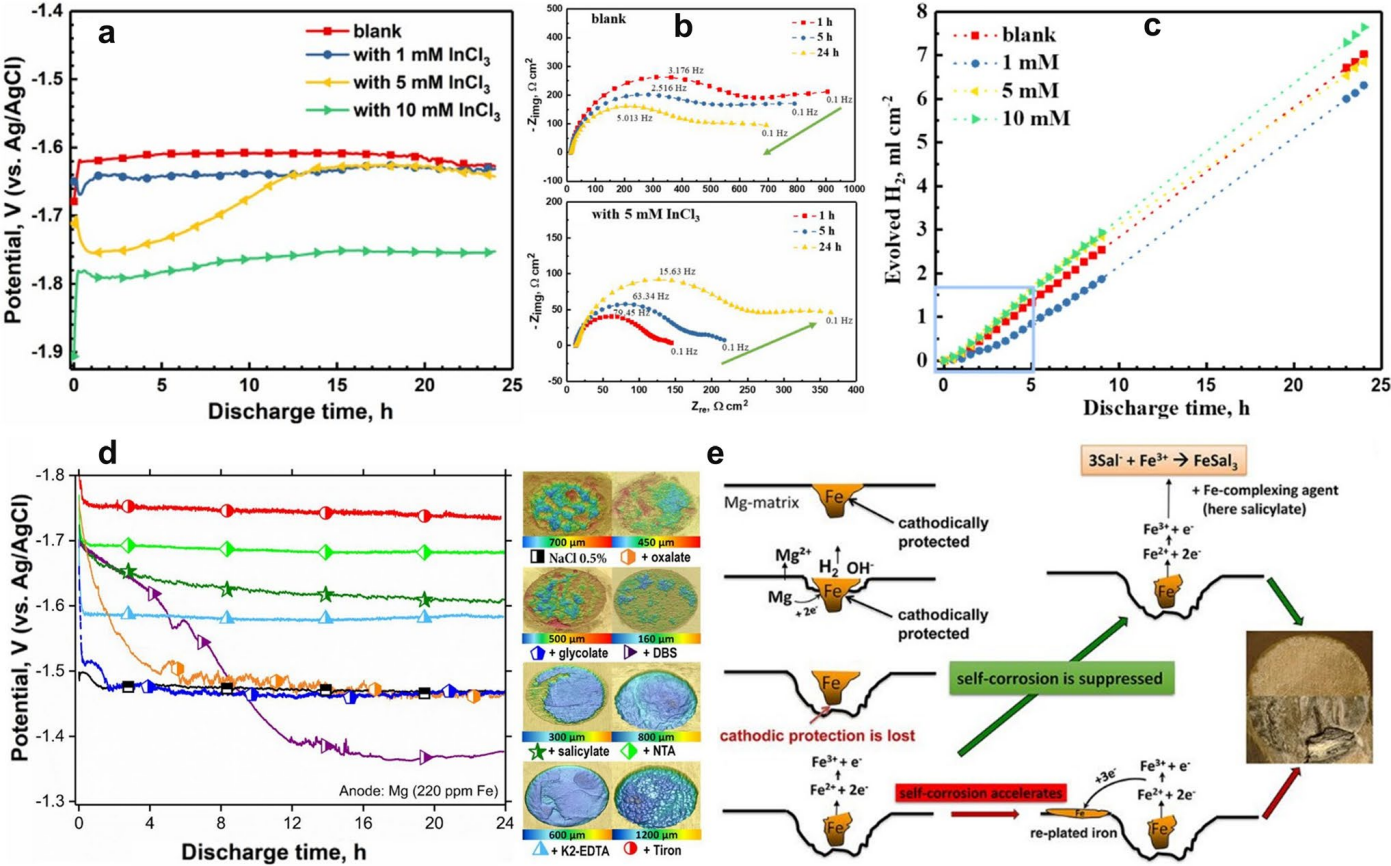
**a** Half-cell discharge curves, **b** EIS plots, and **c** real-time hydrogen evolution test of Mg-0.15Ca anode at 1 mA cm^–2^ in 3.5 wt% NaCl with and without InCl_3_. Reproduced with permission from Ref. [86] Copyright 2021, Elsevier. **d** Half-cell discharge curves of commercial purity Mg (220 ppm Fe) anode in iron-complexing agents-containing electrolytes and 3D maps (right) show the surface morphology of the anodes after the tests. **e** Re-plating of impurity particles accelerates self-discharge of the anode. Interruption of the re-plating mechanism allows for the suppression of related anode fouling; Discharge curves obtained at 0.5 mA cm^−2^ constant current in the half-cell setup with 0.5% NaCl electrolyte and 0.5% NaCl containing additives (0.05 M). Reproduced with permission from Ref. [27] Copyright 2018, Springer Nature

In general, the formation of protective physical barrier surface films is a widely adopted strategy for inhibiting the self-discharge of Mg alloys [79–85]. However, the formation of high-resistance protective films to suppress anodic self-discharge typically results in a trade-off: a reduction in discharge voltage due to increased internal resistance. This section systematically summarizes various discharge mechanisms involving additives that enhance the performance of aqueous Mg batteries without forming protective physical barriers. As shown in Fig. 8a, in the work of Wang et. al. [86], InCl_3_ with different concentrations was selected as electrolyte additives for Mg-0.15Ca anode. The average DPs of Mg-0.15Ca anode in either 1, 5 or 10 mM InCl_3_-containing electrolytes were better than that in the 3.5 wt% NaCl electrolyte. This improvement is attributed to hydrolysis of In^3+^, acidifying either bulk or that and local pH and the formation of a thin In(OH)_3_ layer, which exhibits relatively low film impedance (Fig. 8b). Besides that, the addition of 1 mM InCl_3_ also enhanced UE of Mg-0.15Ca anode. This discharge mechanism suggests that the In(OH)_3_ layer does not serve as a dense physical barrier against the penetration of corrosive species; however, it can lead to a low self-discharge of the Mg anode, possibly by mitigating the HER (Fig. 8c) [87]. As shown in Fig. 8d, Höche et al. [27] introduced a series of iron-complexing agents as additives in 3.5% NaCl electrolyte for commercial purity (CP) Mg anode (220 ppm Fe), ultimately significantly increasing the UE of Mg anode. Moreover, optical images reveal that the surfaces of CP-Mg anodes after discharge in electrolytes containing iron-complexing agents remain clean. As shown in Fig. 8e, the mechanism is that the iron-complexing additives can inhibit self-discharge by impeding the redeposition of Fe particles originating from Fe^2+/3+^ ions released from the present impurities in Mg onto the surface of the anode. In conclusion, while the role of Fe^2+/3+^ complexing agents in suppressing NDE has been described above, the pathways by which other electrolyte additives—such as Mg^2+^ complexing agents, pH buffers, and corrosion inhibitors—suppress the NDE remain to be elucidated.

#### Role of Additives on ORR and Chunk Effect

**Fig. 9:**
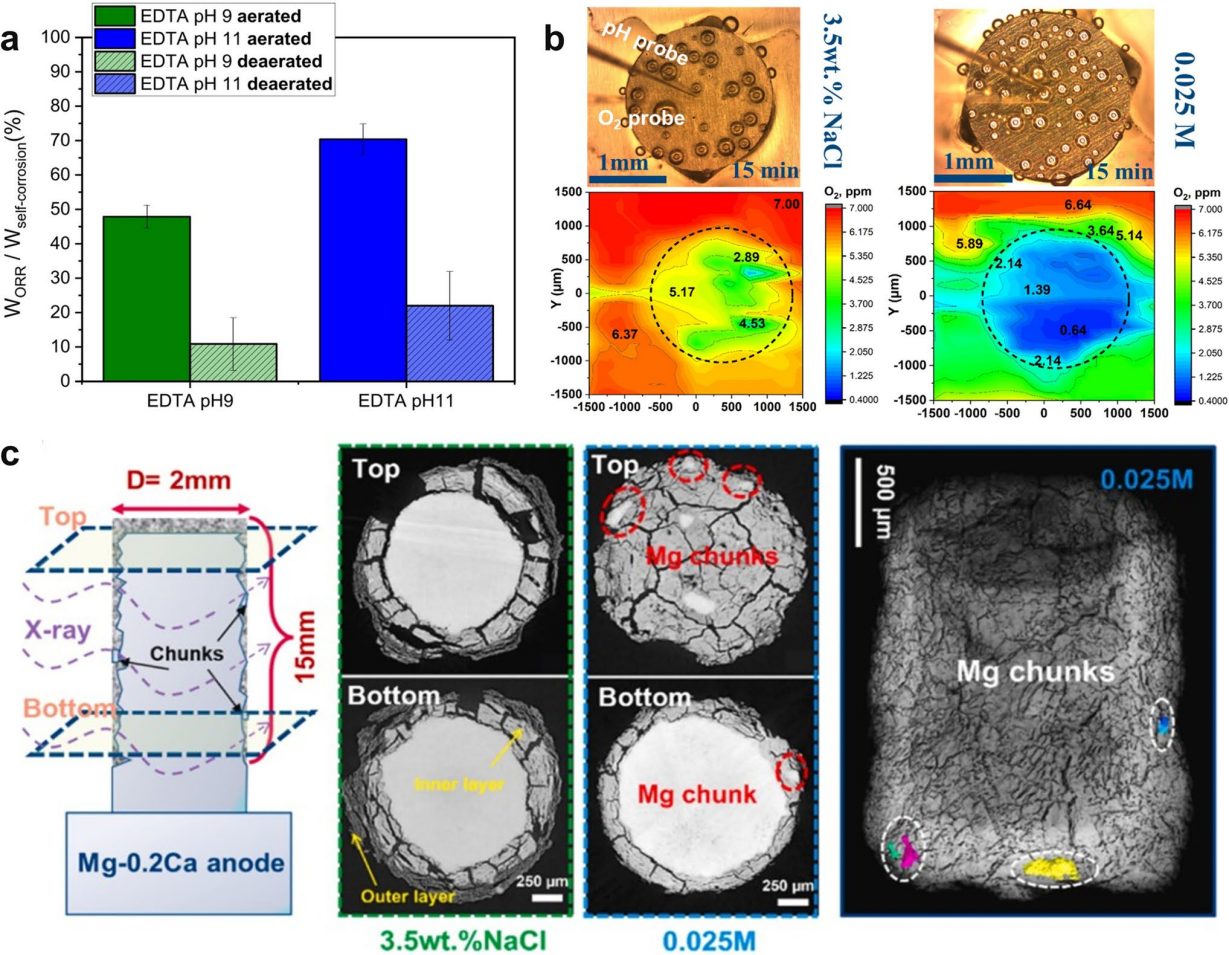
**a** Contribution of W_ORR_ to W_self-discharge_ after 24h of discharge of CP-Mg in aerated and deaerated 3.5 wt% NaCl containing 0.1 M EDTA with initial pH values of 9.0 and 11.0. Reproduced with permission from Ref. [89] Copyright 2022, Elsevier. **b** Scanning localized in-operando microprobe techniques. 2D distribution of concentration of DO, recorded during discharge at 5 mA cm-2, 50 µm above round Mg-0.2Ca anode shown in optical micrographs. **c** Cross-section of Mg-0.2Ca anodes after discharge in 3.5 wt% NaCl and 3.5 wt% NaCl with 0.025 M glutamate electrolytes obtained by synchrotron radiation microtomography and the corresponding 3D rendering from SRµCT. 3D rendering highlighting the identified chunks in the sample discharged in 0.025 M glutamate and (applied current density: 5 mA cm^−2^; Discharge time: 6 h). Reproduced with permission from Ref. [38] Copyright 2025, Elsevier

In addition to the self-discharge mechanism related to NDE, additional self-discharge mechanism related to ORR and chuck effect should be explored. Although the ORR, as a secondary parasitic cathodic reaction at the Mg anode, has often been overlooked during discharge in NaCl-based electrolyte systems—mainly due to the formation of discharge product films that inhibit oxygen diffusion [88]—its contribution in electrolytes containing additives might be more significant. The presence of additives may alter film morphology or composition, potentially enabling ORR and chunk effect, challenging the assumption of its negligible role. This is because some electrolyte additives can maintain a clean anode surface. Moreover, in practical applications, a flowing electrolyte system ensures a sufficient supply of dissolved oxygen, thereby creating a favorable environment for the ORR. For instance, as shown in Fig. 9a, Vaghefinazari et al. [89] demonstrated a considerable ORR contribution during the discharge process after introducing EDTA into the 3.5 wt% NaCl electrolyte, with ORR accounting for 70% of the anode mass loss due to self-discharge. Besides that, as shown in Fig. 9b, Wu et al. [38] proved that the increment of glutamate concentration increases the ORR, which contributed to significant weight loss of Mg-0.2Ca anode. As a result, under discharge in a 3.5 wt% NaCl blank electrolyte, the contribution of the ORR on Mg anode is indeed negligible, primarily because the rapid formation of Mg(OH)_2_ & MgO discharge products forms a thick surface layer that limits oxygen transport to the Mg surface. However, when additives are introduced, the situation changes substantially. Many additives promote removal, dissolution, or thinning of the discharge product film, thereby increasing the anode potential. Once this protective layer is weakened, dissolved oxygen in the electrolyte can reach the Mg surface more readily, leading to a non-negligible ORR contribution. In such cases, ORR becomes an important competing cathodic process and can significantly influence both the DP and the UE. In the same work, as shown in Fig. 9c, the volume of metallic Mg chunks in the discharge film after discharge in 0.025 M glutamate-containing electrolyte were quantified by synchrotron radiation microtomography. The chunk effect accounted for a non-negligible proportion of the anode weight loss. Therefore, it is essential to call for future work on aqueous Mg batteries to carefully consider the impact of ORR on the anode UE.

**Fig. 10:**
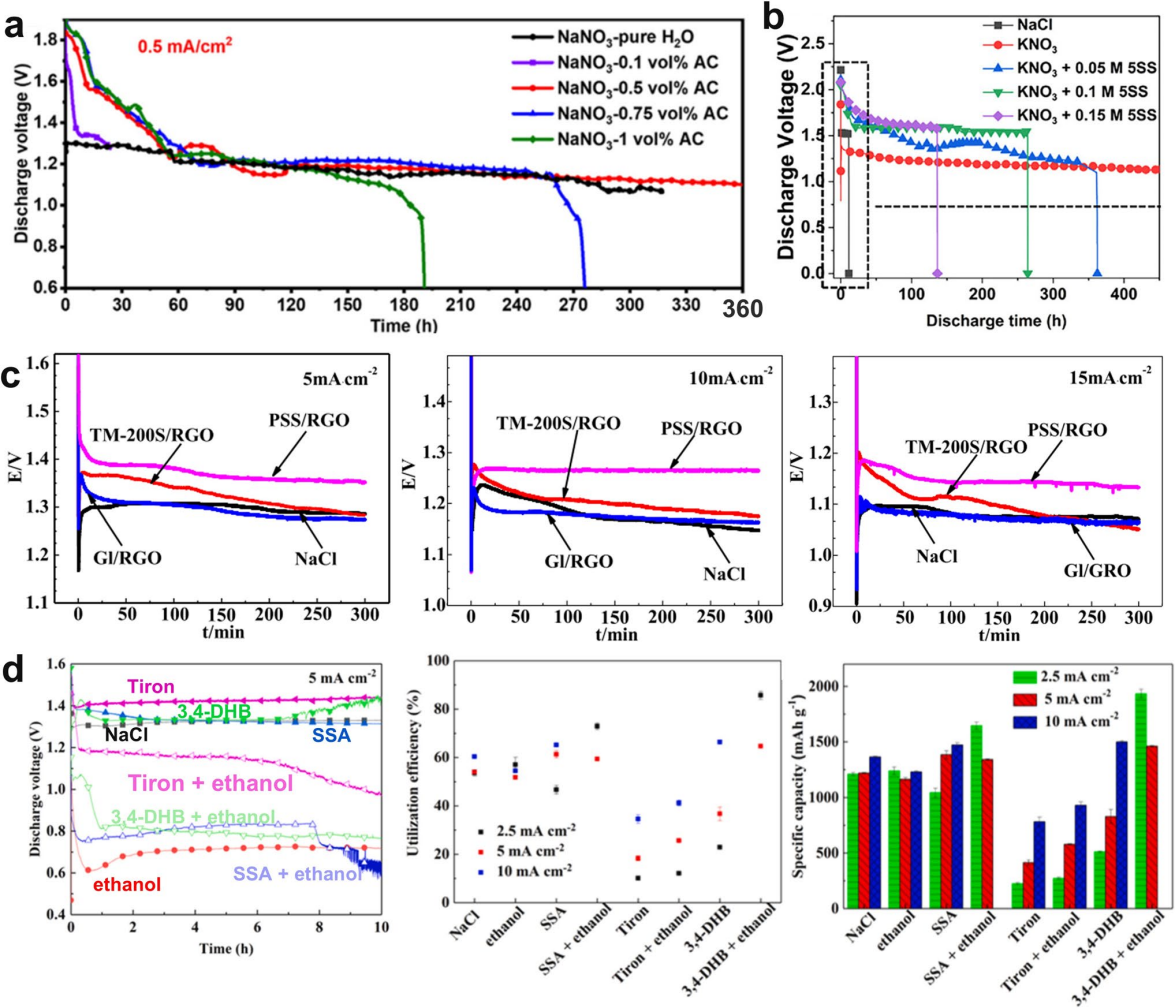
**a** Influence of different concentrations of acetic acid in NaNO_3_–H_2_O electrolyte for long discharge time in full Mg–air battery. Reproduced with permission from Ref. [90] Copyright 2024, Elsevier. **b** Discharge curves of full Mg–air batteries obtained in NaCl, KNO_3_ electrolytes without and with different concentrations of 5SS at discharge density of 0.5 mA cm^−2^. Reproduced with permission from Ref. [90] Copyright 2024, Elsevier. **c** Discharge curves of Mg–air batteries in 3.5 wt% NaCl, 0.5 g L^−1^ PSS/RGO + 3.5 wt% NaCl, 0.5 g L^−1^ TM-200S/RGO + 3.5 wt% NaCl and 0.5 g L^−1^ Gl/RGO + 3.5 wt% NaCl solutions. Reproduced with permission from Ref. [91] Copyright 2022, Elsevier. **d** Discharge voltage, UE, and specific capacity of Mg–air battery in solutions with kinds of additive at different current densities. Reproduced with permission from Ref. [39] Copyright 2024, Elsevier

Although most organic additives in Mg–air batteries are primarily introduced to regulate Mg anode processes such as corrosion suppression and interfacial film formation, their influence on the air cathode should not be neglected. Dissolved organic species may migrate through the electrolyte and reach the air electrode, where they can adsorb on catalytic active sites and carbon-based gas diffusion layers, leading to partial blockage of oxygen reduction reaction (ORR) pathways. In addition, these additives may alter the wettability and surface tension of the electrolyte, thereby affecting oxygen transport and increasing the risk of cathode flooding or gas transport limitations. While systematic studies on additive crossover effects in Mg–air systems remain limited compared with anode-focused investigations, evidence from aqueous metal–air batteries suggests that electrolyte composition can significantly impact cathode polarization and ORR kinetics. Therefore, a holistic understanding of electrolyte–electrode interactions is essential for evaluating the overall equilibrium and performance of Mg–air battery systems.

## Design of Mixed Electrolyte Additives

Based on the discharge mechanism of Mg anodes in individual-component electrolytes, additives can optimize key parameters such as cell voltage and anode UE. However, achieving simultaneous improvement of these parameters is often not possible with single additives alone. Using a mixture of additives with complementary functions can enhance both UE and DP simultaneously and, in some cases, lead to a synergistic effect. Moreover, the composition of such mixtures can be tailored to suit a wide variety of Mg-based anodes. In this review, we present an overview of high-efficiency mixed electrolyte additives and the strategic design principles behind their development. Until now, to the best of our knowledge, only a few research works related to electrolyte additive mixtures for aqueous Mg batteries. For example, Snihirova et al. [40] investigated the discharge performance of 15 Mg anodes of varying compositions, whose UE values were below 60% after discharge at 5 mA cm^−2^ in a 3.5% NaCl electrolyte. After mixing the additives of inorganic NaNO_3_ and organic sodium salicylate, the UE of CP-Mg anode exceeds 80% and the discharge voltage is higher than in its single additive-containing electrolytes. However, when the commercially pure magnesium (CP–Mg) anode changed to HP-Mg anode, the mixed additive fails to improve the discharge voltage of HP-Mg and contributed to a lower UE than that of blank electrolyte. This phenomenon may be attributed to the HP-Mg with small amount of Fe impurity content, which makes the Fe-complexing additive (sodium salicylate) lose its intended functionality, leading to diminished synergistic effects. A similar approach involving mixtures of inorganic and organic components was reported by Światowska et al. [66, 90], as shown in Fig. 10a, b. They employed combinations of KNO₃ with sodium 5-sulfosalicylate and NaNO_3_ with acetic acid as electrolyte additive mixtures for Mg–air batteries. In both studies, the enhancement in discharge voltage was mainly attributed to the ability of 5-sulfosalicylate and acetic acid to complex with Mg^2^⁺ and regulate the precipitation rate of discharge products. However, as already demonstrated, this improved voltage output was accompanied by a decrease in anode UE compared with that obtained in electrolytes containing only nitrate. In addition, as shown in Fig. 10c, Ma et al. [91] achieved simultaneous improvements in DP and UE of Mg–air batteries with an AP65 anode through the incorporation of both organic components of polystyrene sulfonate and reduced graphene oxide (PSS/RGO). However, the cell voltages are below 1.36 V and the anodic utilizations are below 58%, which are relatively low values. In recently published work, as shown in Fig. 10d, Zhou et al. [39] proposed a new strategy that utilizes mixtures of ethanol and complexing agents as electrolyte additives to improve the discharge performance of Mg–air batteries. Results reveal that a mixture of 30 vol% ethanol and 0.1 M complexing agent, especially 0.1 M 3,4-dihydroxybenzoic acid (3,4-DHB) achieved a very high pure Mg anodic UE of 85.7%. However, its discharge voltage is much lower than that of the discharge voltage with 0.1 M complexing agent added, and even lower than 3.5% blank NaCl. The drop of cell voltage might be attributed to the high impedance of discharge layer.

**Fig. 11:**
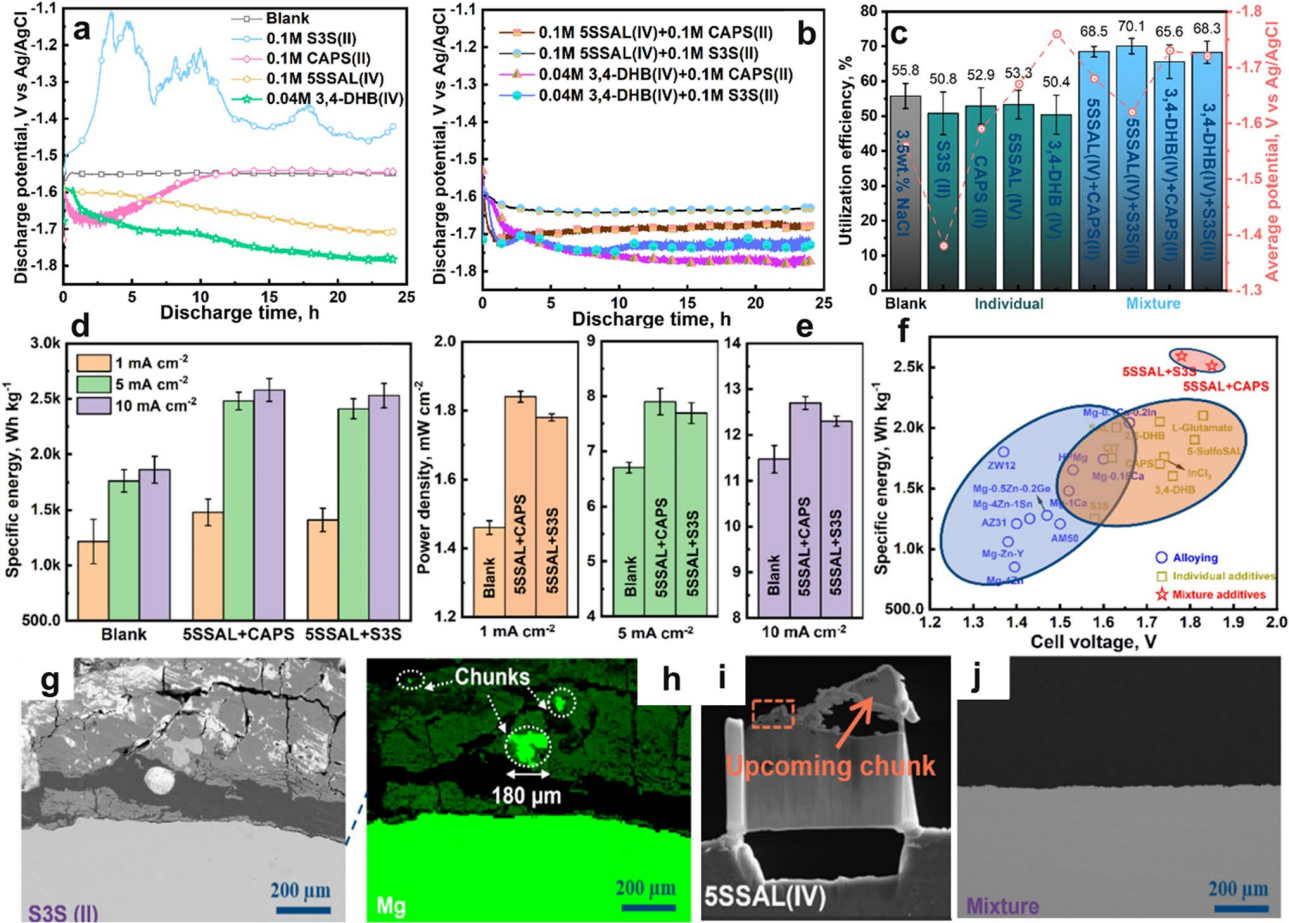
**a** Half-cell DP curves of Mg-0.2Ca anode in individual additive electrolytes and **b** in binary additive mixtures at 5 mA cm^−2^. **c** Corresponding UE and average DP of Mg-0.2Ca anode. **d** Specific energy of Mg-0.2Ca anode in individual additive electrolytes and in binary additive mixtures at 5 mA cm^−2^. **e** Power density of Mg-0.2Ca anode in individual additive electrolytes and in binary additive mixtures at 1, 5, and 10 mA cm^−2^. **f** Specific energy and cell voltage of the Mg–air battery compared with the previously reported works, including alloying, individual, and additive mixtures (applied current density is 5 mA cm^−2^). **g**–**j** Cross-sectional morphologies obtained in the individual additive-containing electrolytes, mixture additive-containing electrolyte. Reproduced with permission from Ref. [50] Copyright 2025, Elsevier

**Fig. 12:**
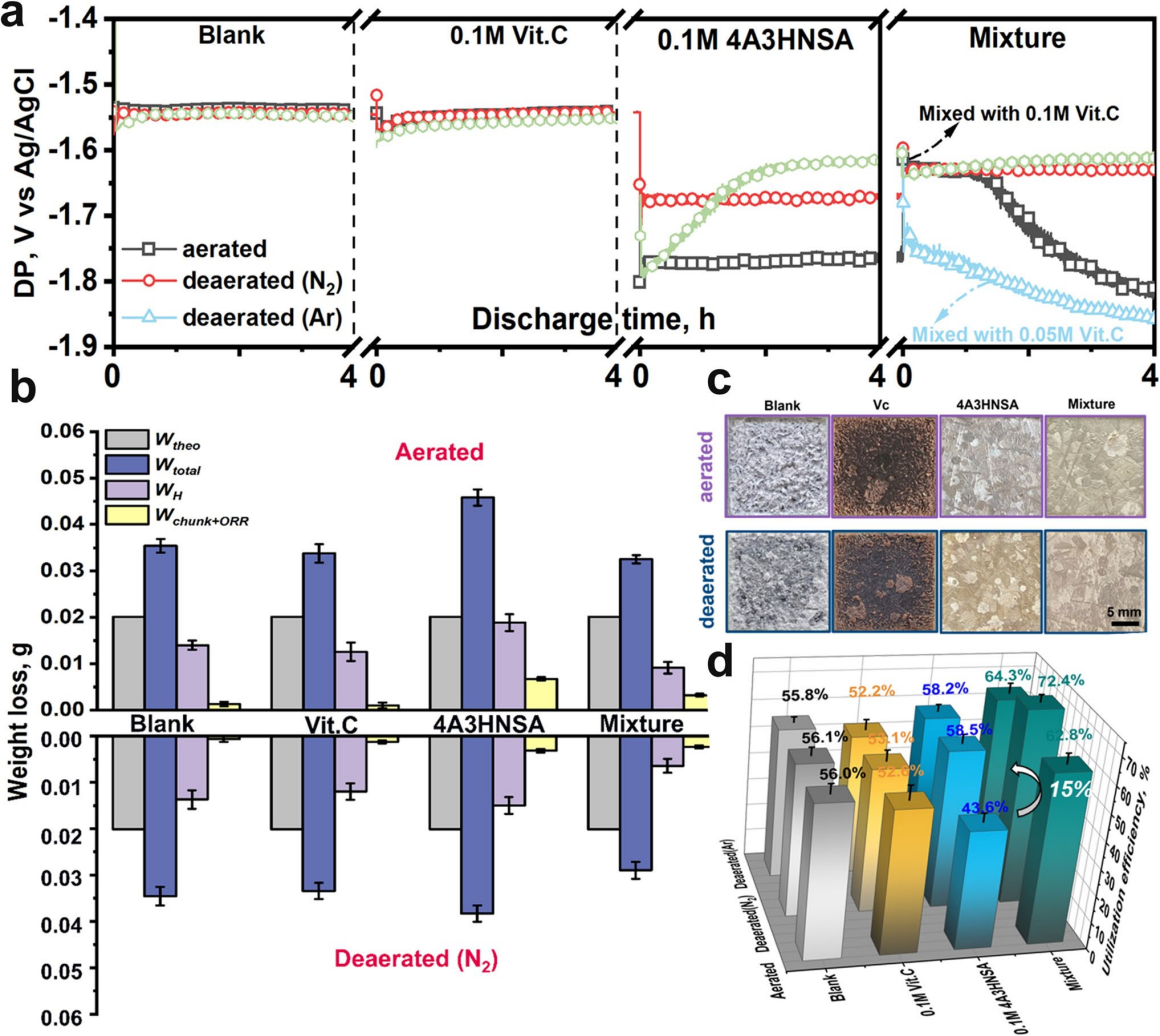
**a** Discharge performance of Mg-0.2Ca anode half-cell batteries under 5 mA cm.^−2^ for 4 h. **b** Calculated weight loss. **c** Optical images of Mg-0.2Ca anodes after discharge in blank, individual additive, and mixed additive electrolytes. **d** Anodic utilization efficiency (UE) of Mg-0.2Ca anodes in blank, individual additive, and binary mixture of electrolyte additives. (*Aerated* indicates discharge in cases when no additional aeration or oxygenation was done, whereas *Deaerated* indicates discharge after O_2_ removal via N₂ or/and Ar purging). Reproduced with permission from Ref. [92]

Building on the mechanistic insights of individual additives, several strategies for developing high-performance electrolyte additive mixtures are proposed herein. First, as demonstrated in our published work [50], electrolyte additives can be classified according to their underlying discharge-modifying mechanisms, and mixtures can be designed to exploit the complementarity between these mechanisms. As shown in Fig. 11, the binary electrolyte additive mixtures system was composed of two distinct individual electrolyte additive systems, each with a specific role. One serves predominantly as a buffer, such as S3S and CAPS, whose DP become worse with increasing discharge time, along with thicker discharge films and more Mg chunks formation. The other functions as Mg^2+^ complexing agents, such as 5SSAL and 3,4-DHB, whose DP is enhanced, while their binding ability to Mg^2+^ also increases the risk of forming Mg chunks. After combining them, the DP, UE, specific energy, and power density of Mg-0.2Ca anode all achieved enhanced at the same time. The underlying mechanism is that the mixed electrolyte additives can suppress the HER, ORR, and chunk effect of Mg anode. Second, as shown in Fig. 12a–c, based on our recent findings [92], Mg^2+^ complexing agents can keep the Mg anode surface clean and a good DP. By conducting a control experiment involving the deaerated electrolyte, we found that the cleaner surface can intensify ORR-driven self-discharge and ultimately lead to a low anode UE (Fig. 12c). Therefore, incorporating a deoxidizing component, such as L-ascorbic acid (Vit. C) into these Mg^2+^ complexing agents-based additives is recommended to mitigate ORR-induced self-discharge, improve anode UE, and maintain a stable and good cell voltage. Continually, a promising future direction is to employ machine learning models trained on large experimental datasets to intelligently screen and identify effective combinations of mixed electrolyte additives. The main advantage of this approach is its ability to accelerate discovery and uncover non-obvious additive synergies. However, it requires a sufficiently large and diverse database, careful feature selection, and robust validation to avoid overfitting and ensure practical applicability.

## Summary of Electrolyte Additives

Table 1 lists representative electrolyte additives studied for Mg–air batteries and their reported effects on anode discharge performance. This review has a dual purpose: to highlight trends in both single and binary additive development, and to provide a structured dataset to guide future machine-learning-based screening of additive combinations.

**Table 1:** Classification and summary of published electrolyte additives and their effects on the discharge performance of Mg anodes

Anodes	Concertation/additives	Applied current density (mA cm^−2^)	Cell voltage, V/ DP, V vs. Ag/AgCl		Other performance	References
*Work of individual*						
Mg	0.05 M HNa_2_PO_4_	3.2	−1.54 V_Ag/AgCl_		HE rates: 0.37 µmol cm^−2^ min^−1^; Mg^2+^ Faradaic efficiency: 31%	[19]
Mg	1 g L^−1^ WSG	1.5	~ 0.6 V		Specific capacity: 1031 mAh g^−1^	[77]
Pure Mg	2.5 mM Decyl glucoside	2 5 8	1.39 V 1.36 V 1.25 V		Specific capacity: 2054 mAh g^−1^ Specific capacity: 1987 mAh g^−1^ Specific capacity: 1608 mAh g^−1^	[34]
Mg (220 ppm Fe)	0.05 M DBS	0.5	−1.38 V_Ag/AgCl_		UE: 48.6% pH after 24 h of discharge: 6.8	[27]
	0.05 M NTA		−1.68 V_Ag/AgCl_		UE: 6.5% pH after 24 h of discharge: 8.3	
	0.05 M Oxalate		−1.47 V_Ag/AgCl_		UE: 21.8% pH after 24 h of discharge: 9.9	
	0.05 M Salicylate		−1.61 V_Ag/AgCl_		UE: 27.2% pH after 24 h of discharge: 9.6	
	0.05 M Tiron		−1.75 V_Ag/AgCl_		UE: 5.0% pH after 24 h of discharge: 7.3	
	0.05 M K_2_-EDTA		−1.58 V_Ag/AgCl_		UE: 7.8% pH after 24 h of discharge: 7.2	
	0.05 M Glycolate		−1.47 V_Ag/AgCl_		UE: 21.6% pH after 24 h of discharge: 9.8	
CP-Mg	0.05 M Tiron	0.5	−1.70 V_Ag/AgCl_		pH after 24 h of discharge: 7.3 UE:34.9% (0.005 M Tiron)	[76]
	0.05 M NTA		−1.69 V_Ag/AgCl_		pH after 24 h of discharge: 8.3	
	0.05 M Sulfosalicylic acid		−1.66 V_Ag/AgCl_		pH after 24 h of discharge: 10.1	
	0.05 M 8-HQ-5-SA		−1.65 V_Ag/AgCl_		pH after 24 h of discharge: 7.2	
	0.05 M Salicylic acid		−1.63 V_Ag/AgCl_		pH after 24 h of discharge: 9.7	
	0.05 M Citric acid		−1.61 V_Ag/AgCl_		pH after 24 h of discharge: 9.2	
	0.05 M DTPA		−1.59 V_Ag/AgCl_		pH after 24 h of discharge: 7.4	
	0.05 M EDTA		−1.58 V_Ag/AgCl_		pH after 24 h of discharge: 7.3	
	0.05 M HEDTA		−1.57 V_Ag/AgCl_		pH after 24 h of discharge: 7.5	
	0.05 M TTHA		−1.54V_Ag/AgCl_		pH after 24 h of discharge: 7.4	
	0.05 M Glycolic acid		−1.53 V_Ag/AgCl_		pH after 24 h of discharge: 8.8	
	0.05 M Norleucine		−1.51 V_Ag/AgCl_		pH after 24 h of discharge: 8.7	
	0.05 M Glycolic acid		−1.47 V_Ag/AgCl_		pH after 24 h of discharge: 9.8	
	0.05 M Hypoxanthine		−1.44 V_Ag/AgCl_		pH after 24 h of discharge: 8.3	
Mg-0.15Ca	10 mM InCl_3_	1 5 10	1.74 V 1.42 V 1.31 V		Specific energy: 2.26 KWh kg^−1^ Specific energy: ~ 1.75 KWh kg^−1^ Specific energy: ~ 1.6 KWh kg^−1^	[86]
Mg-0.15Ca	0.1 M CIT	1 5 10	~ 1.63 V ~ 1.38 V ~ 1.26 V		UE: 64.7%; Specific energy: ~ 3.0 KWh kg^−1^ UE: 60.5%; Specific energy: ~ 1.75 KWh kg^−1^ UE: 56.5%; Specific energy: ~ 1.7 KWh kg^−1^	[36]
	0.1 M SAL	1 5 10	~ 1.64 V ~ 1.39 V Drop below 1 V		UE: 46.3%; Specific energy: ~ 2.3 KWh kg^−1^ UE: 62.9%; Specific energy: ~ 2.25 KWh kg^−1^ UE: 58.8%; Specific energy: ~ 1.5 KWh kg^−1^	
	0.1 M 2,6-DHB	1 5 10	~ 1.39 V ~ 1.36 V ~ 1.25 V		UE: 37.8%; Specific energy: ~ 1.7 KWh kg^−1^ UE: 65.0%; Specific energy: ~ 2.0 KWh kg^−1^ UE: 67.2%; Specific energy: ~ 1.85 KWh kg^−1^	
	0.1 M 5-sulfoSAL	1 5 10	~ 1.82 V ~ 1.42 V ~ 1.32 V		UE: 24.6%; Specific energy: ~ 1.25 KWh kg^−1^ UE: 56.1%; Specific energy: ~ 2.0 KWh kg^−1^ UE: 61.7%; Specific energy: ~ 1.9 KWh kg^−1^	
	0.1 M 3,4-DHB	1 5 10	~ 1.77 V ~ 1.57 V ~ 1.28 V		UE: 9.0%; Specific energy: ~ 0.5 KWh kg^−1^ UE: 35.6%; Specific energy: ~ 1.5 KWh kg^−1^ UE: 62.3%; Specific energy: ~ 1.85 KWh kg^−1^	
Mg-0.15Ca	0.1 M 8-HQ-5-SA	5	−1.55 V_Ag/AgCl_		After discharge 4 h, UE: 59.7%	[41]
	0.1 M Salicylic acid		−1.6 V_Ag/AgCl_		After discharge 4 h, UE: 59%	
	0.1 M l-Lysine		−1.59 V_Ag/AgCl_		After discharge 4 h, UE: 56.7%	
	0.1 M 2-Hyhdroxy-5-nitrobenzoic acid		−1.52 V_Ag/AgCl_		After discharge 4 h, UE: 51%	
	0.1 M Fumaric acid		−1.44 V_Ag/AgCl_		After discharge 4 h, UE: 52.1%	
	0.1 M ACES		−1.72 V_Ag/AgCl_		After discharge 4 h, UE: 40.8%	
	0.1 M Malonic acid		−1.53 V_Ag/AgCl_		After discharge 4 h, UE: 46.4%	
	0.1 M 5-Aminosalicylic acid		−1.55 V_Ag/AgCl_		After discharge 4 h, UE: 46.7%	
	0.1 M Diglycolic acid		−1.55 V_Ag/AgCl_		After discharge 4 h, UE: 47.9%	
	0.1 M 5-Methyl-2nitrobenzoic acid		−1.54 V_Ag/AgCl_		After discharge 4 h, UE: 41.1%	
	0.1 M TAPSO		−.8 V_Ag/AgCl_		After discharge 4 h, UE: 32.9%	
	0.1 M Citric acid		−1.56 V_Ag/AgCl_		After discharge 4 h, UE: 46.5%	
	0.1 M Maleic acid		−1.46 V_Ag/AgCl_		After discharge 4 h, UE: 42.7%	
	0.1 M Ascorbic acid		−1.55 V_Ag/AgCl_		After discharge 4 h, UE: 41.2%	
	0.1 M CHES		−1.84 V_Ag/AgCl_		After discharge 4 h, UE: 31.9%	
	0.1 M EDTA		−1.61 V_Ag/AgCl_		After discharge 4 h, UE: 35.4%	
	0.1 M l-Cysteine		−1.63 V_Ag/AgCl_		After discharge 4 h, UE: 36.5%	
	0.1 M Thiosalicylic acid		−1.57 V_Ag/AgCl_		After discharge 4 h, UE: 41.8%	
	0.1 M Oxalic acid		−1.38 V_Ag/AgCl_		After discharge 4 h, UE: 45.2%	
	0.1 M 5-Sulfosalicylic acid		−1.65 V_Ag/AgCl_		After discharge 4 h, UE: 39.2%	
	0.1 M Tricine		−1.61 V_Ag/AgCl_		After discharge 4 h, UE: 32.3%	
	0.1 M Triethyenetetramine		−1.56 V_Ag/AgCl_		After discharge 4 h, UE: 38.1%	
	0.1 M Bicine		−1.71 V_Ag/AgCl_		After discharge 4 h, UE: 26.1%	
	0.1 M Tris		−1.54 V_Ag/AgCl_		After discharge 4 h, UE: 38.9%	
	0.1 M 3,4-Dimethylbenzoic acid		−1.5 V_Ag/AgCl_		After discharge 4 h, UE: 38.9%	
	0.1 M HEPES		−1.85 V_Ag/AgCl_		After discharge 4 h, UE: 28.6%	
	0.1 M EGTA		−1.61 V_Ag/AgCl_		After discharge 4 h, UE: 25.9%	
	0.1 M NTA		−1.63 V_Ag/AgCl_		After discharge 4 h, UE: 29.4%	
	0.1 M 5-Methylsalicylic acid		−1.54 V_Ag/AgCl_		After discharge 4 h, UE: 35.9%	
	0.1 M 4-Hydroxybenzoic acid		−1.58 V_Ag/AgCl_		After discharge 4 h, UE: 38.7%	
	0.1 M 3,4-DHB		−1.64 V_Ag/AgCl_		After discharge 4 h, UE: 33.8%	
	0.1 M 2,3-DHB		−1.61 V_Ag/AgCl_		After discharge 4 h, UE: 31.1%	
	0.1 M 2,6-DHB		−1.61 V_Ag/AgCl_		After discharge 4 h, UE: 38.5%	
	0.1 M 3,5-DHB		−1.65 V_Ag/AgCl_		After discharge 4 h, UE: 39.4%	
	0.1 M DTPA		−1.61 V_Ag/AgCl_		After discharge 4 h, UE: 25.7%	
	0.1 M 3-Methylsalicylic acid		−1.27 V_Ag/AgCl_		After discharge 4 h, UE: 33.8%	
	0.1 M Tiron		−1.68 V_Ag/AgCl_		After discharge 4 h, UE: 16.4%	
	0.1 M 2-Aminoethanesulfonic acid		−1.86 V_Ag/AgCl_		After discharge 4 h, UE: 24.5%	
	0.1 M 1,3,4-Thiadiazole-2,5-dithiol dipotassium salt		−1.64 V_Ag/AgCl_		After discharge 4 h, UE: 40.6%	
	0.1 M Sodium-4-hydroxybenzenesulfonate		−1.77 V_Ag/AgCl_		After discharge 4 h, UE: 25.6%	
	0.1 M Hydroquinonesulfonic acid		−1.65 V_Ag/AgCl_		After discharge 4 h, UE: 44.1%	
	0.1 M 3-Amino-4-hydroxybezenesulfonaic aicd		−1.85 V_Ag/AgCl_		After discharge 4 h, UE: 29%	
	0.1 M BES		−1.81 V_Ag/AgCl_		After discharge 4 h, UE: 33%	
	0.1 M MOPS		−1.81 V_Ag/AgCl_		After discharge 4 h, UE: 29.6%	
	0.1 M Sodium-1-butanesulfonate		−1.54 V_Ag/AgCl_		After discharge 4 h, UE: 41.7%	
	0.1 M TES		−1.84 V_Ag/AgCl_		After discharge 4 h, UE: 30.1%	
	0.1 M MES		−1.56 V_Ag/AgCl_		After discharge 4 h, UE: 44.2%	
	0.1 M Sulfanilic acid		−1.54 V_Ag/AgCl_		After discharge 4 h, UE: 42.2%	
	0.1 M 3-Nitrobezenesulfonic acid		−1.53 V_Ag/AgCl_		After discharge 4 h, UE: 35.1%	
	0.1 M 1,4-Benzenedimethanol		−1.55 V_Ag/AgCl_		After discharge 4 h, UE: 41.4%	
	0.1 M 1,4-Bis(2-hydroxyethyl)piperazine		−1.57 V_Ag/AgCl_		After discharge 4 h, UE: 46.2%	
	0.1 M 1,2,4-Aminonaphtholsulfonic acid		−1.78 V_Ag/AgCl_		After discharge 4 h, UE: 39.8%	
	0.1 M 1-Pentanesulfonic acid		−1.55 V_Ag/AgCl_		After discharge 4 h, UE: 61.1%	
	0.1 M 4-Aminobutyric aicd		−1.69 V_Ag/AgCl_		After discharge 4 h, UE: 42.5%	
	0.1 M 4-Isopropylbenzoic acid		−0.74 V_Ag/AgCl_		After discharge 4 h, UE: 38.7%	
	0.1 M CAPS		−1.61 V_Ag/AgCl_		After discharge 4 h, UE: 49.9%	
	0.1 M Gallic acid		−1.62 V_Ag/AgCl_		After discharge 4 h, UE: 39.4%	
	0.1 M Triethanolamine		−1.56 V_Ag/AgCl_		After discharge 4 h, UE: 44.4%	
	0.1 M 2-Methoxybenzoic acid		−1.54 V_Ag/AgCl_		After discharge 4 h, UE: 50.7%	
Mg-0.2Ca	0.1 M 2,4-Dihydroxyquinoline	5	−1.53 V_Ag/AgCl_		UE: 53%	[42]
	0.1 M 1-Naphthoic acid	5	−1.57 V_Ag/AgCl_		UE: 55%	
	0.1 M 2,3-Dihydroxynaphthalene	0.5 1	1.80 V 1.69 V		UE: 80.1%; Specific energy: 3.37 KWh kg^−1^ Specific capacity: 1927 mAh g^−1^ UE: 85.0%; Specific energy: 3.21 KWh kg^−1^ Specific capacity: 1876 mAh g^−1^	
AZ31	0.1 wt% Li_2_CrO_4_	10	1.02 V		UE: 68.3%	[65]
AZ31	0.02 M Na_3_PO_4_ ·12H2O	10	1.03 V		Specific capacity: 1068 mAh g^−1^	[64]
	0.02 M NaVO_3_	10	1.07 V		Specific capacity: 1538 mAh g^−1^	
AZ31B	0.1 wt% WDNMoS_2_	1.5	~ 0.8 V		Specific capacity: 1170 mAh g^−1^	[93]
*Mixtures of electrolyte additives*						
Pure Mg	30 vol% Ethanol	2.5 5 10	−1.61 V_Ag/AgCl_ −1.59 V_Ag/AgCl_ −1.53 V_Ag/AgCl_	UE: 57.0%; Specific energy: 1.24KWh kg^−1^ UE: 51.8%; Specific energy: 1.16 KWh kg^−1^ UE: 54.5%; Specific energy: 1.23 KWh kg^−1^		[39]
	0.1 M SSA	2.5 5 10	−1.80 V_Ag/AgCl_ −1.71 V_Ag/AgCl_ −1.62 V_Ag/AgCl_	UE: 46.7%; Specific energy: 1.04 KWh kg^−1^ UE: 61.3%; Specific energy: 1.38 KWh kg^−1^ UE: 65.2%; Specific energy: 1.47 KWh kg^−1^		
	30 vol% Ethanol + 0.1MSSA	2.5 5 10	−1.68 V_Ag/AgCl_ −1.61 V_Ag/AgCl_ ^–^	UE: 72.9%; Specific energy: 1.65 KWh kg^−1^ UE: 59.4%; Specific energy: 1.34 KWh kg^−1^ -		
	0.1 M 3,4-DHB	2.5 5 10	−1.83 V_Ag/AgCl_ −1.73 V_Ag/AgCl_ −1.63 V_Ag/AgCl_	UE: 22.9%; Specific energy: 0.51 KWh kg^−1^ UE: 36.7%; Specific energy: 0.83 KWh kg^−1^ UE: 66.4%; Specific energy: 1.50 KWh kg^−1^		
	30 vol% Ethanol + 0.1 M 3,4-DHB	2.5 5 10	−1.67 V_Ag/AgCl_ −1.62 V_Ag/AgCl_ −	UE: 85.7%; Specific energy: 1.94 KWh kg^−1^ UE: 64.7%; Specific energy: 1.46 KWh kg^−1^ -		
	0.1 M Tiron	2.5 5 10	−1.92 V_Ag/AgCl_ −1.87 V_Ag/AgCl_ −1.79 V_Ag/AgCl_	UE: 10.1%; Specific energy: 0.23 KWh kg^−1^ UE: 18.3%; Specific energy: 0.41 KWh kg^−1^ UE: 34.6%; Specific energy: 0.78 KWh kg^−1^		
	30 vol% Ethanol + 0.1 M Tiron	2.5 5 10	−1.90 V_Ag/AgCl_ −1.83 V_Ag/AgCl_ −1.77 V_Ag/AgCl_	UE: 12.1%; Specific energy: 0.27 KWh kg^−1^ UE: 25.6%; Specific energy: 0.58 KWh kg^−1^ UE: 41.2%; Specific energy: 0.93 KWh kg^−1^		
**CP-Mg**	0.1 M NaNO_3_	5	−1.42 V_Ag/AgCl_	HE rates: 1.00 µmol cm^−2^ min^−1^; UE: 47%		[40]
	0.1 M SAL		−1.16 V_Ag/AgCl_	HE rates: 1.64 µmol cm^−2^ min^−1^; UE: 13%		
	0.1 M NaNO_3_ + 0.1MSAL		−1.50 V_Ag/AgCl_	HE rates: 0.43 µmol cm^−2^ min^−1^; UE: 77%		
Mg-6Al-1In	1% PSS/RGO	10	0.88 V	Specific energy: 1.62 KWh kg^−1^		[94]
AZ91	0.5 g L^−1^Na_3_PO_4_	20	1.150 V	UE: 49.1%		[95]
	0.5 g L^−1^ SDBS		1.141 V	UE: 48.1%		
	0.5 g L^−1^ Na_3_PO_4_ + 0.5 g L^−1^ SDBS		1.134 V	UE: 48.7%		
AZ61	5.0 mM Na_2_SnO_3_	5 10 25 40 55 70 85	−1.60 V_Ag/AgCl_ −1.57 V_Ag/AgCl_ −1.54 V_Ag/AgCl_ −1.47 V_Ag/AgCl_ −1.42 V_Ag/AgCl_ −1.34 V_Ag/AgCl_ −1.23 V_Ag/AgCl_		Specific capacity: 946 mAh g^−1^ Specific capacity: 1175 mAh g^−1^ Specific capacity: 1392 mAh g^−1^ Specific capacity: 1481 mAh g^−1^ Specific capacity: 1448 mAh g^−1^ Specific capacity: 1253 mAh g^−1^ Specific capacity: 1054 mAh g^−1^	[96]
	5.0 mM Na_2_SnO_3_ + 0.1 mM CMCS	5 10 25 40 55 70 85	−1.63 V_Ag/AgCl_ −1.55 V_Ag/AgCl_ −1.52 V_Ag/AgCl_ −1.45 V_Ag/AgCl_ −1.42 V_Ag/AgCl_ −1.36 V_Ag/AgCl_ −1.26 V_Ag/AgCl_		Specific capacity: 1009 mAh g^−1^ Specific capacity: 1323 mAh g^−1^ Specific capacity: 1494 mAh g^−1^ Specific capacity: 1582 mAh g^−1^ Specific capacity: 1641 mAh g^−1^ Specific capacity: 1676 mAh g^−1^ Specific capacity: 1325 mAh g^−1^	
Mg-0.2Ca	0.1 M 5-SulfoSAL + 0.1 MCAPS	1 5 10	1.84 V 1.58 V 1.27 V		Specific energy: 1.48 KWh kg^−1^ Power density: 1.84 mW cm^−2^ Specific energy: 2.48 KWh kg^−1^ Power density: 7.9 mW cm^−2^ Specific energy: 2.58 KWh kg^−1^ Power density: 12.7mW cm^−2^	[50]
	0.1 M 5-SulfoSAL + 0.1 MS3S	1 5 10	1.78 V 1.48 V 1.23 V		Specific energy: 1.41 KWh kg^−1^ Power density: 1.78 mW cm^−2^ Specific energy: 2.42 KWh kg^−1^ Power density: 7.4 mW cm^−2^ specific energy: 2.53 KWh kg^−1^ Power density: 12.3 mW cm^−2^	
	0.04 M 3,4-DHB + 0.1 M CAPS	5	−1.74 V_Ag/AgCl_		UE: 67.0%	
	30.04 M 3,4-DHB + 0.1 M S3S	5	−1.72 V_Ag/AgCl_		UE: 65.0%	

## Summary and Outlook

Electrolyte additives acting as corrosion inhibitors, Mg^2+^/Fe^2+/3+^ complexing agents, pH buffers, and so on, can modify the electrolyte composition, which plays a crucial role in the discharge performance of aqueous Mg batteries, as the discharge product deposition and Mg dissolution kinetics at the anode surface are closely tied to the solution environment. With the assistance of individual electrolyte additives, the specific energy density of aqueous Mg batteries can readily exceed 3.0 kWh kg^−1^ [42]. Given that individual electrolyte additives that can simultaneously enhance both DP and UE are rare, mixtures electrolyte additives were proposed based on an in-depth understanding of the discharge mechanisms within the Mg anode/electrolyte interface system and limitations of various individual additives. Herein, two different methods for designing electrolyte additives mixtures are summarized.

(I) Regulating the interfacial reactivity of individual additives to suppress self-discharge while preventing the formation of dense discharge products: The explored individual additives, such as corrosion inhibitors, usually promote the growth and formation of denser discharge product films that sometimes can inhibit HER but result in a low DP and severe chunk effect. In contrast, Mg^2+^ complexing agents exhibit strong interfacial reactivity, which promotes continuous dissolution of surface layers and partial removal of nano- and micro-scale Mg chunks. This leads to an elevated DP but inevitably reduces anode UE. Properly selecting and mixing these two types of individual additives can boost discharge properties of Mg anode, preventing issues caused by either excessive or insufficient additive activity, which can lead to inhomogeneous discharge and reduce the chunk effect. This design approach for electrolyte additive mixture has been demonstrated. It simultaneously promotes superior DP and UE in aqueous Mg batteries, generally increasing the specific energy to above 2.5 Wh kg^−1^.

(II) Using oxygen scavengers, e.g., ascorbic acid in electrolyte mixtures design: By utilizing this strategy, more chemically active additives, that can effectively remove thick discharge films, are used in combination with ascorbic acid. This mixture helps to inhibit the ORR of Mg anode by removing dissolved O_2_ in aqueous electrolyte and mitigate the chunk effect on the Mg anode at the same time. In conclusion, individual additives can exhibit synergistic effects when combined, because each component compensates for the limitations of the others. Such mixtures address multiple issues simultaneously, including discharge product film formation, the chunk effect, and ORR-induced self-discharge. This enables the full-cell voltage and specific energy of aqueous Mg batteries to easily exceed 1.8 V at 1 mA cm^−2^ and 2.5 kWh kg^−1^ at 5 mA cm^−2^. Beyond developing new electrolyte additive mixtures, the two systems discussed above still require further extensive experimentation to identify combinations that optimize not only voltage and specific energy, but also stability, compatibility, and long-term discharge performance.

**Fig. 13:**
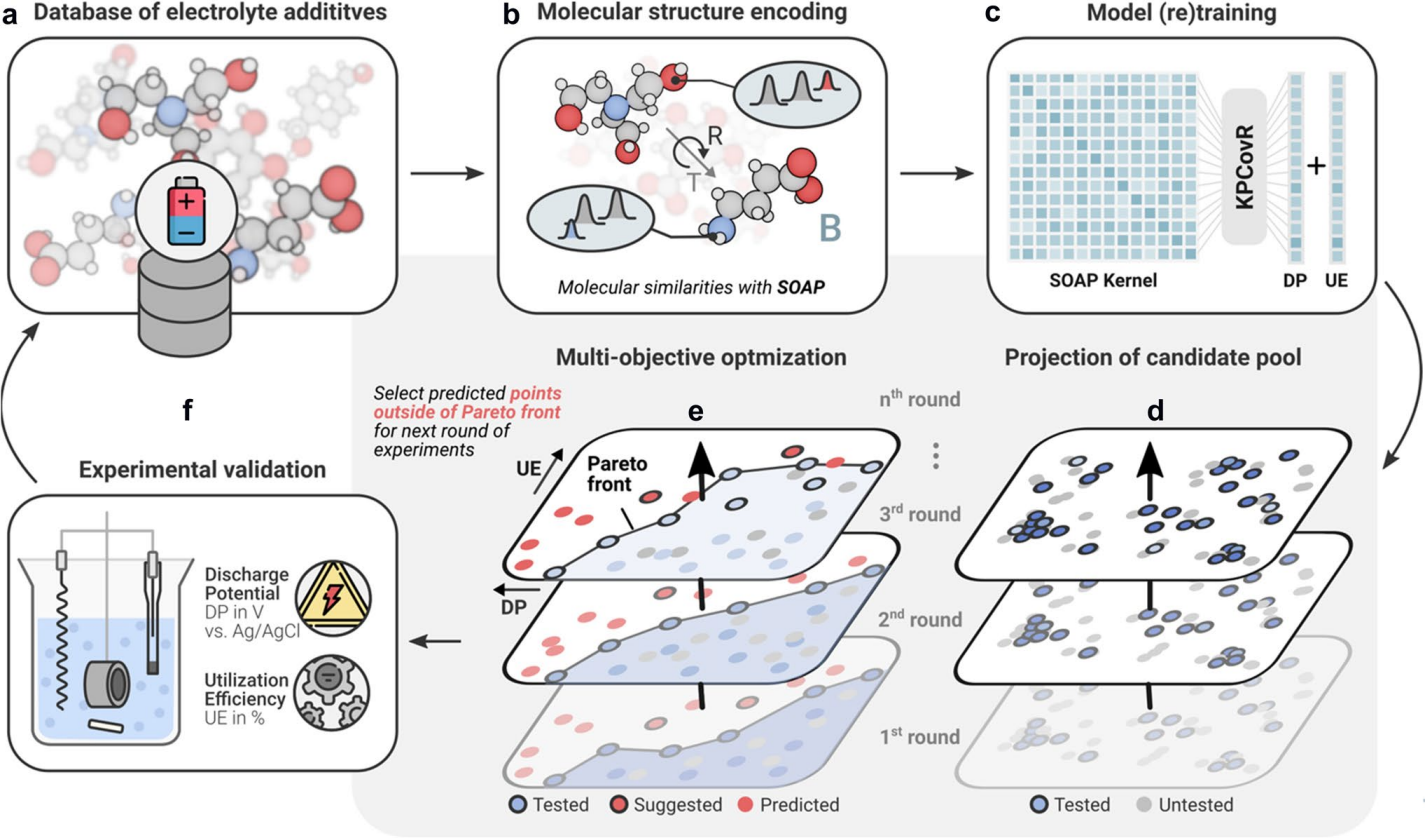
Schematic illustration of the active learning-based adaptive experimental design workflow. **a** Starting from an experimental seed dataset of electrolyte additives for Mg–air batteries. **b** First, the structure of all compounds is encoded using the SOAP approach. **c** Resulting SOAP kernel is used as input for a KPCovR model using the experimentally measured DP and UE values as target. **d** Trained model is used to predict the DP and UE of yet untested candidates from a commercial database. **e** Based on the predictions, promising test candidates are selected using multi-objective optimization. Therefore, structures predicted to lie outside of the Pareto front spanned by the compounds of the training set are considered. **f** After experimental validation, the loop is closed, and the initial database is extended to serve as basis for the next iteration. Reproduced with permission from Ref. [42] Copyright 2025, Elsevier

With the rapid growth of data-intensive applications, the integration of large-scale database systems with machine learning (ML) pipelines has become increasingly attractive [97–99]. In particular, we note that simulating the competitive adsorption of multiple organic species on metallic Mg surfaces remains challenging due to the large configurational space, complex intermolecular interactions, and the dynamic nature of the electrochemical interface. Current studies mainly rely on density functional theory (DFT)-based adsorption energy calculations, ab initio molecular dynamics, and increasingly, machine-learning-assisted simulations to improve sampling efficiency and interpret competitive adsorption behaviors. A representative and reliable database not only facilitates scalable model training but also ensures timely access to high-quality features for real-time inference [100, 101]. As shown in Fig. 13, with the assistance of ML, electrolyte additive 2,3-dihydroxynaphthalene was discovered after already the third loop of ML model predictions. Remarkably, this additive boosted the specific energy of the Mg-0.2Ca anode to 3.37 kWh kg^−1^ and the specific capacity to 1927 mAh g^−1^, approaching the theoretical value of 2205 mAh g^–1^. Inspired by recent advances in other metal-based battery research, the adoption of high-throughput robotic testing platforms in place of manual experimentation is expected to provide substantial potential and value for the future development of aqueous Mg–air batteries. For instance, Noh et al. [102] developed an automated workflow combining high-throughput experimentation with active learning to enhance the solubility of redox-active molecules in organic solvents, demonstrating a generalizable strategy for accelerated materials discovery. Similarly, Dave et al. [103] integrated a custom-built robotic system (“Clio”) with a Bayesian optimization planner (“Dragonfly”) to autonomously identify six fast-charging non-aqueous electrolytes within 42 experiments over two days—achieving a six-fold speed-up over random search. These candidates were further validated in pouch cells, showing improved fast-charging performance compared to a baseline electrolyte. Nevertheless, to date, no studies have been reported in the field of Mg-based batteries that integrate high-throughput robotic testing platform with large-scale data generation and machine-learning-driven analysis in the article context. Based on this, the employment of high-throughput robotic testing platforms, combined with dedicated effort, could boost the throughput and accuracy of experimental testing, as part of cyclical ML workflow in the future, ultimately achieving fully automated and efficient autonomous additive discovery. It is especially relevant for synergistic electrolyte additives, when the potential compositional space becomes nearly infinite. Further, setting up models or even quantum chemical simulations to efficiently predict the optimal initial conditions, such as initial pH, concentration, discharge time, and applied current becomes realistic. Given the limitations of single electrolyte additives of aqueous Mg batteries, novel computational approaches for binary and multicomponent mixture calculations of electrolyte additives should also be developed in the future.
